# Biophysical Mechanistic Modelling Quantifies the Effects of Plant Traits on Fire Severity: Species, Not Surface Fuel Loads, Determine Flame Dimensions in Eucalypt Forests

**DOI:** 10.1371/journal.pone.0160715

**Published:** 2016-08-16

**Authors:** Philip Zylstra, Ross A. Bradstock, Michael Bedward, Trent D. Penman, Michael D. Doherty, Rodney O. Weber, A. Malcolm Gill, Geoffrey J. Cary

**Affiliations:** 1 Centre for Environmental Risk Management of Bushfires, Biological Sciences, University of Wollongong, Wollongong, NSW, Australia; 2 School of Ecosystem and Forest Sciences, The University of Melbourne, Creswick, VIC, Australia; 3 Fenner School of Environment and Society, Australian National University, Acton, ACT, Australia; 4 Physical, Environmental and Mathematical Sciences, University of NSW ADFA, Canberra, ACT, Australia; The Ohio State University, UNITED STATES

## Abstract

The influence of plant traits on forest fire behaviour has evolutionary, ecological and management implications, but is poorly understood and frequently discounted. We use a process model to quantify that influence and provide validation in a diverse range of eucalypt forests burnt under varying conditions. Measured height of consumption was compared to heights predicted using a surface fuel fire behaviour model, then key aspects of our model were sequentially added to this with and without species-specific information. Our fully specified model had a mean absolute error 3.8 times smaller than the otherwise identical surface fuel model (p < 0.01), and correctly predicted the height of larger (≥1 m) flames 12 times more often (p < 0.001). We conclude that the primary endogenous drivers of fire severity are the species of plants present rather than the surface fuel load, and demonstrate the accuracy and versatility of the model for quantifying this.

## Introduction

Plants may affect forest fire behaviour by influencing the quantity and flammability of surface fuel, the three dimensional structure of the forest, and through the flammability of their live parts. The effect of variation in dominant species on surface fine litter load is well established [1] and evidence of species effects on flammability is accumulating [2–5]. The importance of forest structure to fire behaviour and severity, and the role of species in determining this, has however received less attention. Consequently, although there is broad acceptance of a link between this and stand flammability, only a small number of studies have demonstrated this link for a limited range of structural traits, e.g. [6–8]. Crown fire modelling has led the way in this regard [9,10].

The role of leaf traits in influencing fire behaviour via flammability remains uncertain. While strong evidence exists for the influence of chemistry, moisture and morphology on leaf flammability [11–13] and laboratory studies have found correlations with some observed fire behaviour characteristics [14–16], the outcomes of variation in these leaf attributes has not been mechanistically defined for individual plants [17], and remains largely qualitative and incomplete for forest stands [18]. In some cases, conclusions drawn from observations appear to conflict. Belcher *et al* for example characterised sites with broader leaves as less flammable due to a positive correlation between leaf width and time to ignition [19], while others noted an opposite correlation with site flammability, due to the influence of longer or larger leaves in aerating litter layers [4,5].

Given these uncertainties, it has been argued that fire behaviour models can disregard the influence of certain flammable properties of plant parts. Alexander and Cruz [16] for instance proposed that variations in ignitability produced by different leaf moisture values will be overwhelmed by the larger heat fluxes involved in fires under field conditions, and therefore have little influence on rates of spread. However empirical evidence to support this prediction may be lacking [20]. The mechanisms underpinning this prediction also require further elucidation [21]. Schwilk [22] has argued that scaling from plant traits to ecosystem effects is a fundamental goal of functional ecology, but mechanisms for extrapolating flammable traits of plants to large scales, as encompassed in fire behaviour science, are lacking. Here we address this problem through the presentation and validation of a model that incorporates mechanisms for scaling from flammability of individual leaves to forest stand-level fire behaviour attributes such as flame height.

Our aim was to show that stand-level characteristics of fire behaviour could be successfully predicted on the basis of knowledge of the flammable properties of plant species and the interaction of these properties that arise from considering the three dimensional arrangement of whole plants and their relevant, flammable features, such as leaves and stems. Specifically, we predicted flame height using a modelling approach that accounted for the arrangement of plants and their flammable parts plus the influence of the morphology of these parts on flammability. We then tested predictions derived from the model against field estimates of char height or the height to which leaves are consumed by fire following a wildfire, in a range of forest sites across a mountainous region of south eastern Australia.

### The study

Plant traits can potentially affect fire characteristics at the whole plant and stand scale via several fundamental processes. First, the properties of individual leaves such as size, shape, moisture content and chemical composition [11–13,23] determine their ignitability, combustibility and sustainability [24]. Second, the three-dimensional arrangement and spacing of leaves and fine stems either as litter or as live foliage, will affect the likelihood of fire propagating or spreading through the plant via various mechanisms of drying and heat transfer (radiation, conduction, convection—(radiation, conduction, convection, [9,25]). Third, the three dimensional arrangement and spacing of leaves will affect the nature of environmental influences on ignition and spread of fire among fuel elements via effects on insolation and wind [26–28]. Many fire behaviour models only partially represent these processes and arrangements, if at all. The Rothermel model [29] for example includes some determinants of leaf flammability but models heat transfer using only radiation and without explicitly considering the nature of gaps between individual fuel elements. Australian fire behaviour models based on surface ‘fuel load’ or the weight of fine dead material on the ground surface e.g. [30,31] do not explicitly consider any of these influences, whereas the more recent “Project Vesta” model utilises a small subset of vegetation structure and flammability properties to subjectively rank the flammability of some strata into scores [6].

In this study we tested the hypothesis that flammability properties of leaves will significantly affect characteristics of fire at the scale of a forest stand via the mechanisms listed above. Specifically, we examined how variations in flame propagation and resultant height of flames across multiple strata of plants within forest stands emerge from variations in characteristics of component species and their inherent effects on flammability and the spatial arrangement of leaves. We explored this using a comparative modelling approach to examine the sensitivity of predicted variations in the propagation and dimensions of flames to different representations of the structure and composition of eucalypt forest stands. We compared predictions from the models against ground-measured and remotely-sensed estimates of fire severity, for which we used height of combustion in vegetation as an index of flame dimensions [32]. Char height [33], or the height to which leaves were blackened, has been used as an estimate of flame height in some studies, but post-fire measurements were required immediately after the fire [34]; an option that was not available to us. Scorch height differs from char height in that air temperatures above a fire may be sufficient to kill the cells in leaves without producing char. This is frequently considered in severity measurements [32], but relating it back to flame height modelling requires the use of a scorch height model of unknown reliability.

Our measurements were taken within the perimeter of a major wildland fire which burned across diverse terrain, forest and weather conditions. These comparisons allowed us to test whether incorporation of leaf characteristics of individual species and their effects on ignitability, combustibility, sustainability, heat transfer and wind profiles, improved predictions of flame dimensions.

## Materials and Methods

We conducted our study in two stages to quantify the influence of plant species composition and structural manifestation on forest fire behaviour. First, we proposed and tested a site-based biophysical, mechanistic model of fire behaviour as influenced by the species-driven properties of forest structure and leaf traits, and in the second we analysed the model outputs to identify the conditions under which leaf traits were important determinants of fire behaviour, and which groups of traits had the most influence

### Model description and validation

The Forest Flammability Model [35,36], (FFM) was used to explore effects of vegetation structure and leaf flammability on flame propagation and dimensions. Full code, instructions and data required to replicate this study are available at https://github.com/pzylstra/ffm_cpp/tree/Zylstra2016, and further explanation is provided in S1 Table.

**Fig 1:**
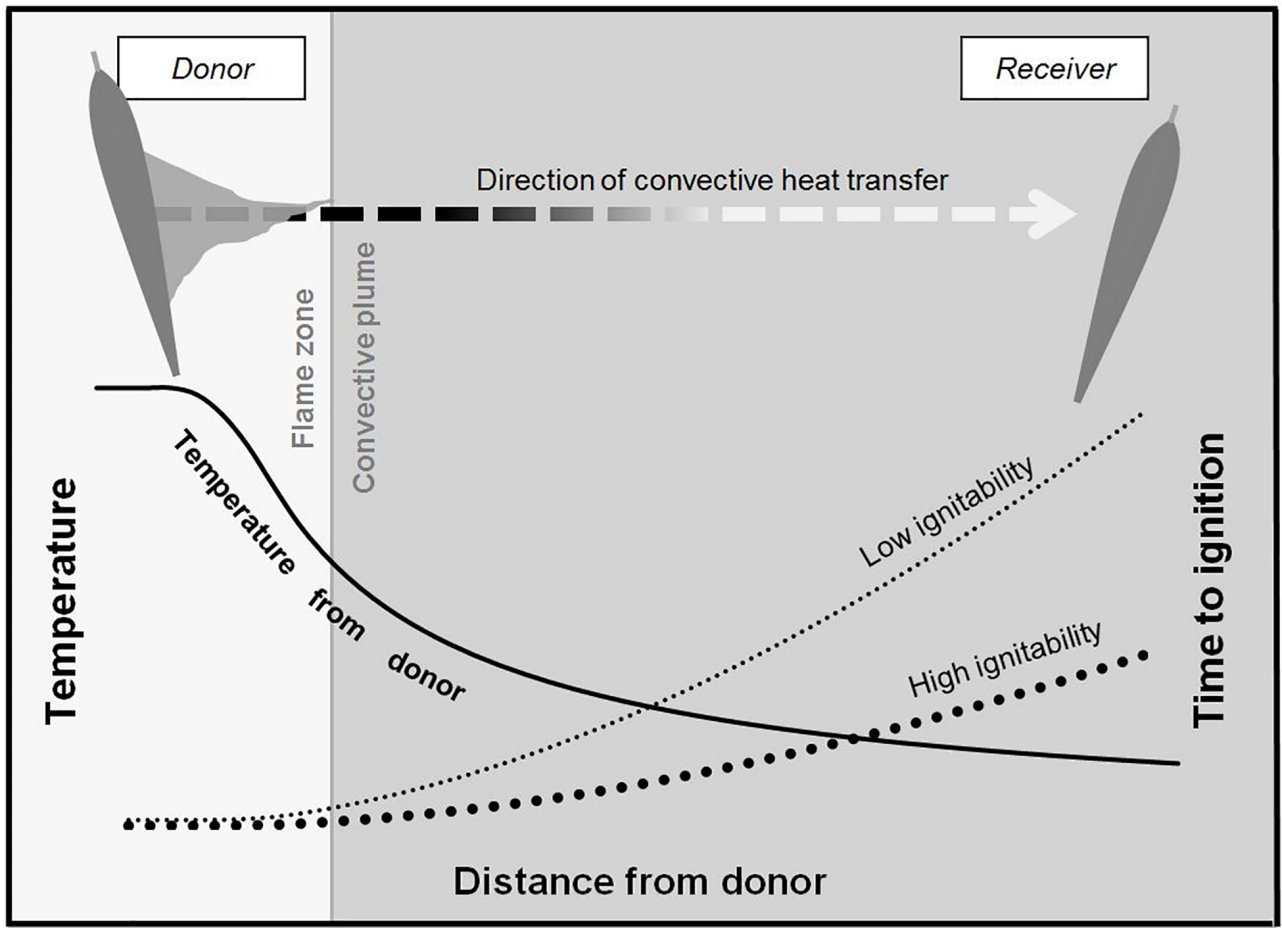
Ignition of a receiver leaf by a burning donor. The flame from the donor produces a convective plume following a direction described by the flame angle (broken arrow), where the temperature of the air in the plume decreases with distance from the donor (solid curve) in a pattern determined by the flame produced from that leaf. The time of heating required for ignition of the receiver increases as the temperature decreases, at a rate determined by the ignitability of the leaf. The plume temperature model is taken from [37], and the time to ignition modelled from [35], where ignitability is a function of plume temperature and the Ignitability Coefficient (IC = leaf moisture (% Oven Dry Weight) * thickness (mm) / number of sides on the leaf).

The FFM predicts fire behaviour from leaf traits and plant structure in the following way:

1. When a leaf ignites it becomes a heat donor, producing a convective plume that decreases in temperature with distance from the source along a vector defined by the flame angle. The heat output of the burning leaf (combustibility) determines the dimensions of the heat plume and the nature of the temperature gradient within it (Fig 1). The duration of burning (i.e. the sustainability of flaming in that leaf) defines the period for which a given temperature is maintained.
2. Leaves ignite if the period of flame duration produced by the donor leaf exceeds the time to ignition for the receiver. This is a function of both the temperature at that point in the plume and the ignitability of the leaf (Fig 1). Leaf ignitability in this study is represented by the Ignitability Coefficient, where IC = leaf moisture (% Oven Dry Weight) * thickness (mm) / number of sides on the leaf. As IC increases, ignitability decreases.
3. This process is repeated at escalating scales from leaf to branch, plant and plant stratum.

Combustibility and ignitability therefore interact in the model to determine the depth of ignition (distance from the flame at which leaves can be ignited), which together with the density of foliage gives the number of leaves ignited for each one-second time step. The angle at which the burning plume intersects a plant crown or stratum defines a plume pathway or potential depth of foliage that can be ignited. This angle is adjusted at each time step as wind speed and flame dimensions affect the flame angle, so that the plume pathway evolves over time. The total number of leaves burning in a time step is the sum of leaves burning in the previous step plus those newly ignited, minus the leaves that have extinguished as determined by their sustainability properties. The resulting flame length for that step is a function of the number of leaves burning, the combustibility of those leaves and physical processes of heat transfer and air entrainment between them.

**Fig 2:**
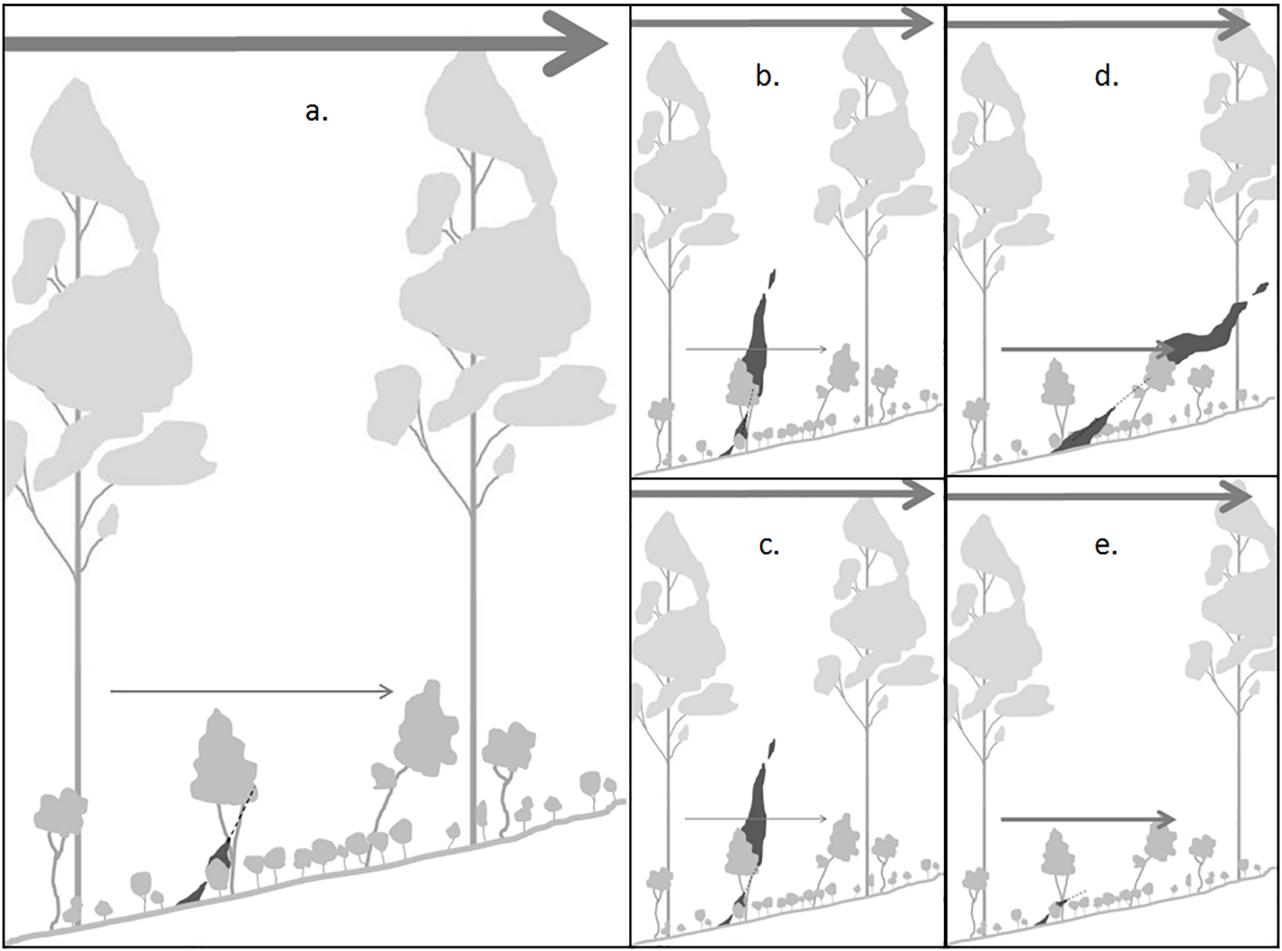
Effects of donor flammability, receiver ignitability and overstorey sheltering on fire severity. Wind speed is shown above and below the canopy by the solid arrows, with thicker and longer arrows showing greater wind speed. The trajectory of convective heat transfer is shown by the broken line. Four scenarios b to e are shown relative to the left scene a. The convective plume produced by the donor plant in *a*. intersects the receiver; however it is insufficient to ignite it. This is changed when in *b* the donor flammability is increased to give a larger flame that ignites the receiver, and in *c* when the donor flame is the same as in *a* but the receiver ignitability is greater. The flammability of the plants in scenarios *d* and *e* is the same as in *a*, but the wider tree spacing has reduced the overstorey sheltering so that the wind speed is greater at the level of the flame. This directs the plume through its neighbouring plants in *d* so that the flame depth is increased and the resulting larger flame ignites the receiver stratum. In scenario *e*, the plume passes over neighbouring shrubs or elevated stratum [7] so that they are not ignited and the flame dimensions remain unchanged from *a* The more acute angle of the plume, however, increases the distance to the receiver stratum so that the heat dissipates and that stratum is even less likely to ignite than in *a*.

In vegetation with multiple plant strata, the severity of a fire expressed as the height of combustion [32] will be determined by the capacity of flames from each stratum to ignite strata above them (Fig 2A). This will be a function of the heat produced by the donor strata, the heat required for ignition by the receiver stratum and the spacing between donor and receiver, as convective heat dissipates over distance. The structure and composition of the forest influences all these processes via the effects of the arrangement and spacing, dimensions, moisture and chemistry of leaves (Fig 2B and 2C).

Plants in strata above a flame but not burning may influence the behaviour of the fire via effects on wind speed in the burning strata. Such effects will be a function of the amount of shelter provided by the overhead foliage, which in the FFM is modelled from the Leaf Area Index (LAI) calculated from leaf size, density on the branch, ramification and spacing of branches in the non-burning strata above the flames, along with the physical size and spacing of plant crowns. These effects on wind may, in turn, affect the angle of the heat plume and thereby the length of the plume pathway, the length of flame produced, and the angle and distance to receiver leaves (Fig 2D and 2E). Thus leaf traits may affect flame dimensions and propagation via the endogenous mechanisms of donor flammability and receiver ignitability, and the exogenous (i.e. environmental effects) mechanism of overstorey sheltering. The model represents both these exogenous and endogenous mechanisms and their effects on flame characteristics and propagation.

The FFM is initiated using a model of surface fire spread that produces a pilot flame to potentially ignite above-ground fuel strata. Species-specific leaf traits are known to affect surface fire behaviour (see introductory discussion), however at this point no model exists that incorporates these effects. In the absence of such a model, this study uses the Burrows model [38] for this purpose, as its development in a controlled laboratory setting was considered to more accurately reproduce the behaviour of flames burning in eucalypt leaf litter alone, as opposed to other leaf litter fire behaviour models developed in field situations [30,31]. When incorporated into the FFM, this model was constrained to the approximate domain of the data from which it was constructed (Figure 7 in [39]), and flame heights were modelled using an angle derived from flame length and wind speed [40] to remain consistent with the rest of the FFM.

#### Model validation

We validated the model by investigating whether variations in structure and species composition of forest stands affected stand-level flame dimensions as predicted. We compared outputs from three modelling treatments with field observations of the height of combustion (height above the ground at which leaves and stems were consumed by fire) as a proxy for flame height in eucalypt forests of south-eastern Australia. The comparative modelling approach enabled us to systematically contrast predictions of stand-level flame dimensions produced by models that treated the representation of fuel with successively increasing levels of complexity. These ranged from surface litter fuel only to full consideration of structural arrangement and species-level leaf dimensions (Tables 1 and 2). This approach provided a test of the null hypothesis that explicit representation of the flammable characteristics of leaves of individual species within a spatial framework does not explicitly improve the capacity to model flame dimensions.

**Table 1:** Fuel and structural parameters used in this study.

Parameter	Collection methods	Stratum			
Surface fuel load	Characterised for each vegetation type by direct measurement at 12% of sites.	9.6–24.3 t.ha^-1^			
		**Near-surface (m;** $\bar{x}$**, range)**	**Elevated (m;** $\bar{x}$**, range)**	**Midstorey (m;** $\bar{x}$**, range)**	**Canopy (m;** $\bar{x}$**, range)**
Plant separation	Calculated from survey for each site	0.7, 0.1–3.6	2.8, 0.7–8.9	9.3, 3.2–26.4	8.4, 0.0–14.1
Crown base centre height	Measured from photograph as proportion of crown height	0.0, 0.0–0.6	0.7, 0.0–2.5	3.2, 0.2–9.5	10.5, 1.3–22.6
Crown base edge height	Measured from photograph as proportion of crown height	0.0, 0.0–0.8	0.9, 0.1–2.7	4.3, 0.3–10.2	11.6, 2.3–25.1
Crown height	Direct measurement in survey for each site	0.3, 0.1–1.0	2.0, 0.2–6.0	8.8, 2.0–15.0	18.2, 8.0–35.0
Crown top edge height	Measured from photograph as proportion of crown height	0.2, 0.0–1.0	1.6, 0.1–4.0	7.1, 1.7–14.6	20.6, 7.0–33.6
Crown width	Measured from photograph as proportion of crown height in all strata except trees, where it was calculated from survey results	0.4, 0.1–1.9	1.5, 0.4–4.2	4.3, 1.5–10.2	4.7, 2.8–6.8

**Table 2:** Leaf traits used to model flammability parameters in this study.

Parameter	Collection methods	Details
Clump diameter	Measured from photograph as proportion of crown height, calculated in m.	Used to calculate the number of leaves burning, given a depth of ignition [35].
Clump separation	Measured from photograph as proportion of crown height, calculated in m.	
Leaf packing	Mean number of leaves per clump within plant crowns, calculated from herbarium and field measurements using empirical relationship in[35]. Unit less	
Endotherm	Unless a measured value was available, most species used a standard temperature of 260°C or 220°C based on aromaticity, as per[35]. Where published values were available, silica-free ash content was used in grasses or ferns as per[41].	The minimum piloted temperature of ignition, determined by leaf chemistry.
Percent dead	Standard values used of 50% for C4 grasses, 0% for other grasses along with most shrubs and trees, visual estimates taken from field visit for some exceptions.	Mean moisture for the foliage is weighted from live and dead moisture contents.
Leaf Form	Taken from published literature or observed	Leaf form (flat or round) and thickness determine the surface area to volume ratio, and together with moisture content determine 90.0% of the time to ignition for sclerophyllous leaves [35].
Leaf thickness	Measured from herbarium specimens and field visits; entered in m	
Leaf width	Measured from herbarium specimens and field visits; entered in m	The cross-section area of the leaf (width * thickness) and leaf moisture account for 73.7% of the flame duration in sclerophyllous leaves with an external heat source [35], leaf length, width and moisture content account for 87.2% of the flame length produced by burning sclerophyllous leaves [35].
Leaf length	Measured from herbarium specimens and field visits; entered in m	
Moisture Content	Standard values of 100%ODW were used for all species except herbs and some mesic species (150%), and very green herbs and mesic species (200%)	

**Fig 3:**
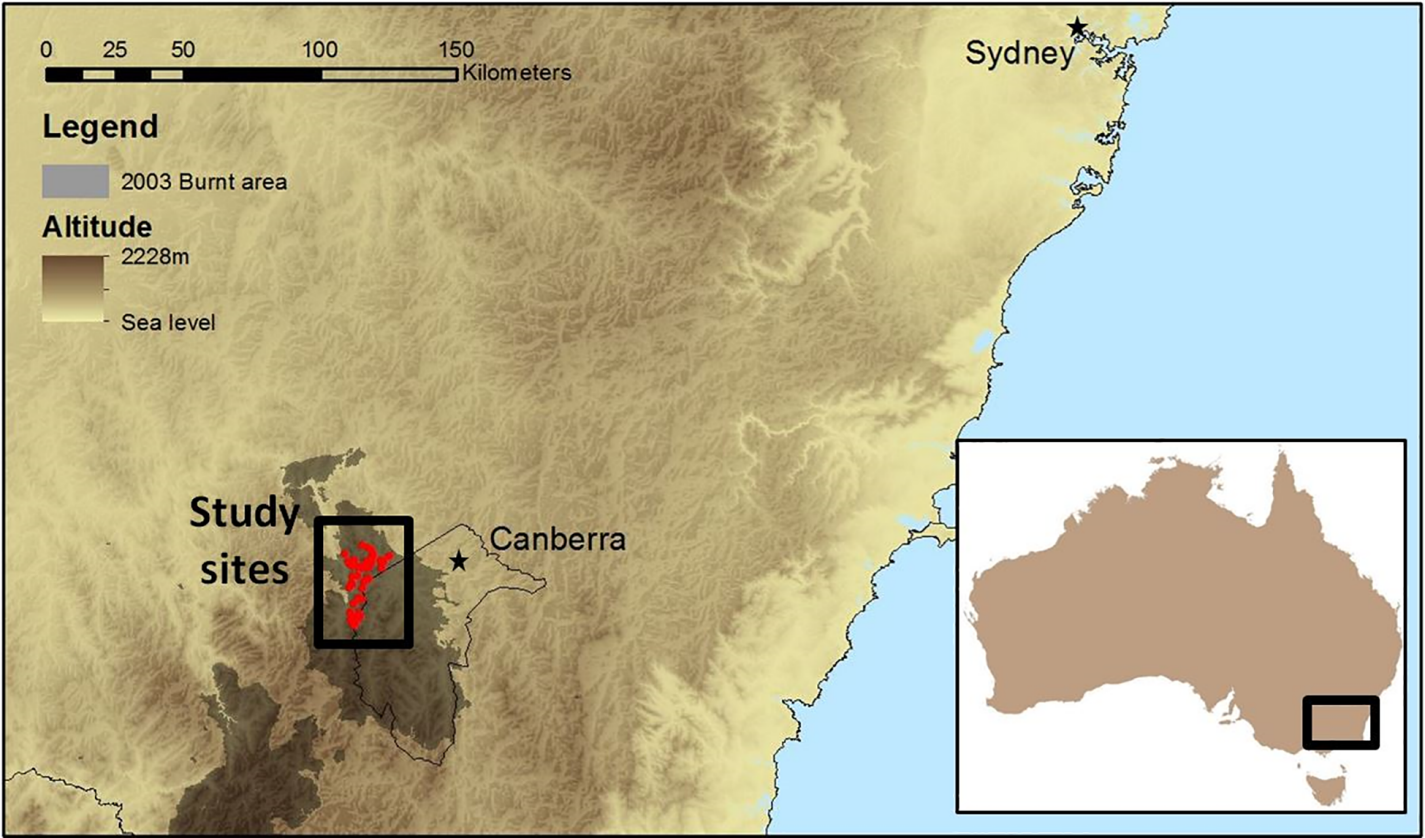
Location of the study sites (open circles) in relation to Canberra, Sydney and the area affected by the 2003 bushfires (shaded).

We tested the null hypothesis by comparing the three sets of predictions against estimates of height of combustion across a range of sites burnt by a major wildland fire in south-eastern Australia (Fig 3). Mountainous terrain and diverse forest types, along with variation in ambient weather conditions at the time of burning, resulted in marked variation in fire behaviour among sites. This provided a wide range of fire severity for the evaluation of predictions representing the influences of vegetation structure and leaf traits affecting fire properties at the stand level.

Three scenarios were modelled to address the hypothesis that vegetation structure and leaf traits are important to fire behaviour; these were:

1. Surface fuel load only (F)–no structural or leaf traits effects of standing plants considered;
2. Surface fuel load and stand structure (FS)–as for (F), but considering structural effects of above ground plants but not effects of leaf traits; and
3. Surface fuel load, stand structure and leaf traits (FSL)–as for (FS), but also considering leaf traits of component species.

#### Study sites

The derivation of various model inputs and estimation of fire severity or height of combustion were made following a large wildfire in January 2003 in the Brindabella Range near Canberra (Fig 1). A subset of 58 sites was sampled from a larger set of 130 that were originally established prior to the fire for a vegetation survey [42–45]. Sites were selected that had been burned at a known time, and for which adequate data existed to provide fuel inputs. The examined sites were situated in eight different montane to sub-alpine classes of forest dominated by *Eucalyptus* species of varying height (8 to 35 m, Table 3). The date and time (morning / afternoon) which the sites were burnt was estimated from maps of fire spread based on on-ground observations and remote sensing (multispectral line-scans from aircraft) (Australian Capital Territory Emergency Services unpublished data). All sites were re-surveyed and photographed after the fire in 2003 [42].

**Table 3:** Forest classes examined in the study, as given in the original survey.

	No. sites	Characteristic species	Moisture class	Height (m)	Time since fire (years)^1^
***E*. *camphora* subsp. *humeana* Open Forest**	1	*E*. *camphora Poa sieberiana Carex appressa*	Wet	25	64
***E*. *dalrympleana—E*. *delegatensis* Tall Open Forest**	3	*E*. *delegatensis E*. *dalrympleana*, *E*. *dives*, *Acacia dealbata*, *Oxylobium ellipticum*, *Coprosma hirtella*, *P*. *sieberiana*	Wet	30–35	51–64
***E*. *dalrympleana—E*. *dives* (+/- *E*. *pauciflora*) Open Forest**	10	*E*. *dalrympleana*, *E*. *robertsonii*, *E*. *pauciflora*, *E*. *dives*, *E*. *macrorhyncha*, *A*. *dealbata*, *A*. *rubida*, *A*. *verniciflua*, *Kunzea ericoides*, *Lomatia myricoides*, *Daviesia mimosoides*, *Cassinia longifolia*, *C*. *aculeata*, *P*. *sieberiana*	Intermediate	10–35	5–64
***E*. *dalrympleana—E*. *fastigata* Tall Open Forest**	7	*E*. *fastigata*, *E*. *dalrympleana*, *E*. *viminalis*, *E*. *delegatensis*, *A*. *melanoxylon*, *A*. *dealbata*, *L*. *myricoides*, *Dicksonia antarctica*, *C*. *aculeata*, *Bedfordia arborescens*, *Olearia stellulata*, *Urtica incisa*, *P*. *sieberiana*, *Pteridium esculentum*, *Polystichum proliferum*, *Blechnum nudum*, *Dichondra repens*, *Hydrocotyle laxiflora*	Wet	20–35	5–25
***E*. *dalrympleana—E*. *pauciflora* Open Forest**	18	*E*. *pauciflora*, *E*. *dalrympleana*, *E*. *viminalis*, *E*. *delegatensis*, *A*. *melanoxylon*, *A*. *dealbata*, *C*. *aculeata*, *Bossiaea foliosa*, *D*. *mimosoides*, *Acrothamnus hookeri*, *Olearia megalophylla*, *Coprosma hirtella*, *Dianella tasmanica*, *P*. *esculentum*, *P*. *sieberiana*, *P*. *phillipsiana*, *Asperula scoparia*, *Dichondra repens*, *Hydrocotyle laxiflora*	Intermediate	8–35	23–64
***E*. *dalrympleana—E*. *robertsonii* Tall Open Forest**	5	*E*. *robertsonii*, *E*. *dives*, *E*. *macrorhyncha*, *E*. *pauciflora*, *E*. *dalrympleana*, *A*. *falciformis*, *A*. *melanoxylon*, *A*. *dealbata*, *C*. *aculeata*, *Bursaria spinosa*, *P*. *esculentum*, *P*. *sieberiana*, *C*. *appressa*, *Acaena novae-zelandiae*	Intermediate	15–35	24
***E*. *dives—E*. *macrorhyncha—E*. *mannifera* (+/- *E*. *rubida*) Open Forest**	10	*E*. *dives*, *E*. *macrorhyncha*, *E*. *mannifera*, *E*. *robertsonii*, *C*. *longifolia*, *C*. *aculeata*, *Phebalium squamulosum*, *D*. *mimosoides*, *Pultenaea juniperina*, *Platylobium formosum*, *Pteridium esculentum*, *Dillwynia phylicoides*, *Rytidosperma pallidum*, *P*. *sieberiana*	Dry	8–15	23–51
***E*. *viminalis—A*. *melanoxylon* Open Forest**	4	*E*. *viminalis*, *E*. *dalrympleana*, *E*. *robertsonii*, *A*. *melanoxylon*, *A*. *dealbata*, *E*. *stellulata*, *Leptospermum grandifolium*, *Coprosma quadrifida*, *C*. *aculeata*, *Pomaderris aspera*, *Poa labillardierei*, *P*. *sieberiana*, *P*. *helmsii*, *C*. *appressa*, *Microlaena stipoides*	Wet	15–30	22–25

^1^ Time since fire refers to time since the last fire previous to 2003

Weather conditions at the time of burning in each site were estimated using gridded weather surfaces for parameters used in the various models (Table 4). Slope and wind speed were calculated relative to the direction of fire spread at each site, so that fire flanks burning at right angles to the wind were given a wind value of zero, and downslope spread was given a negative slope value. Fuel and structural parameters required by the models were calculated from the initial site surveys and photographs (Tables 1 and 2). Leaf characteristics of 53 dominant species used in the FFM were taken from published sources [35,46–49], measurements of herbarium specimens and measurements taken in the field during 2013 at the study sites and in the Australian National Botanic Gardens at Canberra (S5 Table).

**Table 4:** Exogenous factors used in modelling of fire behaviour for this study.

Parameter	Characterisation methods	Range
Slope	Taken from site surveys and adjusted by the angle of fire spread in relation to the terrain	-28° to 21°
Wind direction	Modelled on a 250 m grid using Butler *et al* [50] from wind directions recorded for Cabramurra (63km SW of sites, closest high altitude station) and Canberra (20km E of sites, closest station) weather stations. Lee-slope directions were adjusted for dynamic channelling effects using a terrain filter developed by Sharples *et al* [51].	0°-335°
Wind velocity	Modelled on a 250 m grid using [50]from wind directions recorded for Cabramurra and Canberra weather stations, adjusted for the direction of fire spread relative to the wind.	-21 to 30km/h
Air temperature	Linear interpolation between upper and lower (Cabramurra and Canberra) values based on elevation.	12.5 to 37.6°C
Relative humidity	Linear interpolation between upper and lower (Cabramurra and Canberra) values based on elevation.	13.0 to 63.5%
Dead fuel moisture	Modelled using Gould *et al* [39]	3.6 to 13.7%

#### Field estimation of flame heights

In the absence of definitive evidence, we estimated a range for the flame heights at each site rather than assigning a single value. The minimum was set by the height of combustion, which was measured at the study sites from multiple sources: i.e. remotely sensed difference Normalised Burn Ratio (dNBR,e.g. [32]) and estimates of fine stem consumption from the post-fire survey and photographs in 2003 [42]. In the field survey, a stratum was classed as burnt if both leaves and fine stems were absent at the time of post-fire surveys, and the minimum flame height was derived from either this or the presence of char on smooth barked trees as discussed by Alexander and Cruz [52]. Flame height was estimated as being within the bounds defined by the height of combustion (loss of leaves and fine twigs) and the lowest of either the surveyed scorch height or the base of the next unconsumed stratum. Estimates of fire severity derived from dNBR using remote sensing (i.e. LANDSAT imagery at 25 m resolution) were obtained from a major survey after the fires [53], and where field records and photographs were unclear, the dNBR was used to identify whether elevated and crown strata were burnt or unburnt using the classes in the Barrett survey. “Low” severity indicated 50% burn in the elevated layer and all higher classes indicated complete burn, and “high’ indicated 50% burn of the canopy, so that classes below this were unburnt and those above were completely burnt.

#### Flame height prediction

Predictions of flame height for each site were produced using site-specific exogenous conditions (Table 4) and addressing the three treatments already described. Treatment F was modelled using [38] alone, FS was modelled using the FFM, but leaf traits were averaged into the two groups monocotyledon and dicotyledon and internal structure of plants was not characterised. FSL was also modelled using the FFM, with incorporation of species-specific estimates of leaf traits and using internal structure as summarised in Table 2 and listed in S5 Table.

All treatments used wind speed adjusted for overstorey sheltering by vegetation, and this was summarised for each site using the modelled wind reduction factors (WRF: above-canopy wind speed / wind speed at 1.5 m above ground level) as approximations of the full vertical wind-speed profiles used by the FFM for each site.

The predictions produced from each approach were then compared with the site-based upper and lower limits of flame height to determine whether the inclusion of leaf traits and their arrangement within various strata improved the accuracy of predictions.

The absolute error (AE) of predictions was estimated to identify treatment bias, and the mean absolute error (MAE) to provide a natural and unambiguous measure of model performance [54]. To better identify the nature of treatment errors, the proportion of correct predictions (PCP) was calculated for all sites, and PCP_1_ for a subset of sites where measured flames were at least 1m in height. The MAE, PCP and PCP_1_ were then compared between successive inclusions of structure and leaf traits using paired *t*-tests to determine whether any apparent improvements were statistically significant for those statistics. This approach was chosen in preference to a more standard nested-model comparison, as in addition to identifying the better treatment, it quantified the accuracy of each with statistics that we considered meaningful to model application.

### Sensitivity of flame height to environmental conditions and specific leaf traits

In the second stage, we examined how the inclusion of leaf traits affected the relative importance of the three key mechanisms represented in the FFM (i.e. overstorey sheltering, donor flammability, receiver ignitability) in predicting estimated flame height. This was done by comparing the way that overall flame height changed between the FS and FSL treatments in response to parameters representing the three key processes, along with a range of other influences representing characteristics of the study sites, weather at the time of burning and other measures of vegetation/fuel structure (Table 5).

**Table 5:** Leaf traits driving differences between FSL and FS treatments in this study.

Mechanism	Driving Factors	Effects
Overstorey sheltering	Leaf width, length, separation, branch ramification, clump diameter and clump separation, used to calculate Leaf Area Index^1^ in all plant strata above those burning.	Affects the angle of flames, potentially changing flame length by altering the depth of burning fuel (Fig 3D) or changing the amount of heat received by changing the length of the path to receiver plants (Fig 3E).
Donor flammability	All traits of donor plants	Affects the flame length by changing the flammability of the fuel burning (Fig 3B)
Receiver ignitability	Leaf thickness, moisture content, number of sides (affecting surface area), endotherm, percent dead in receiver plants^2^	Affects the capacity for receiver plants to ignite by changing their ignitability (Fig 3C).

^1^ LAI used to calculate the vertical wind field as per [27,35]

^2^ Ignitability consists of the endotherm and the *IC*, where leaf moisture is the mean of live and dead moisture contents weighted by the percent dead in the plant.

To achieve this, we performed Least Absolute Shrinkage and Selection Operator (LASSO) regression analysis [55] to identify which of the three mechanisms most influenced differences in predicted flame height from the mean and species models. A set of candidate predictors was derived to represent each of the three mechanisms (Table 6); further predictors were added to represent site and environmental influences, giving 12 candidate predictors. All predictors were continuous variables.

**Table 6:** Predictors used in the LASSO regression of change in predicted flame height.

Leaf Traits	
Donor Flammability	
Delta donor Fh	Difference between treatments in modelled donor flame height (m)
Donor Fh_FS	Donor flame height modelled in FS (m)
Receiver Ignitability	
Mean IC_FSL	Average of all ignitability coefficients in FSL
Overstorey Shelter	
Sum LAI_FS	Sum of all LAI values in FS
Delta sum LAI	Difference between treatments in sum of all LAI values
Canopy LAI_FS	Canopy LAI in FS
Delta canopy LAI	Canopy LAI in FSL–canopy LAI in FS

Other Factors	
Other fuel descriptors	
Surface fine fuel	Surface fuel load (t^.^ha^-1^)
Vertical continuity	Proportion of the full crown height composed of plant canopies
Exogenous Factors	
DFMC	Dead fuel Moisture Content (%)
Wind velocity	Wind speed adjusted for direction relative to fire spread (km^.^h^-1^)
Slope	Slope, derived from DEM (degrees)

LASSO regression was used to simultaneously fit models and select those subsets of predictors having most influence, with the coefficients for other predictors being forced to zero. The response variable was predicted flame height under FS minus that under FSL. Additive regression models were fitted using the “glmnet” package [56] for R [57], with the single tuning parameter chosen to either minimize prediction error as estimated by random k-fold cross-validation, or to find the most parsimonious regression model with a prediction error within one standard error of the minimum value. We used the cv.glmnet function with k = 10.

Trial regressions showed that there was substantial variation in the predictors chosen when the fitted model was selected using minimum prediction error (“min” rule) in repeated runs of the cross-validation procedure. In contrast, only one predictor (Delta donor Fh) was present in models selected with the one-standard-error rule (“1se” rule). This indicated a single dominant variable with a number of secondary variables of much less influence, whose inclusion depended on the random allocation of sites to training and test folds during cross-validation. Since our purpose was interpretation rather than prediction, we did not wish to simply discard these secondary variables. To investigate them further we undertook an ensemble regression analysis in which 1000 LASSO models were selected using the “min” rule, and the frequency of inclusion of each predictor in the fitted additive models was recorded along with statistics (mean, upper 97.5% and lower 2.5% quantiles) describing the characteristic regression coefficients for each.

## Results

### Field estimations of flame height

**Fig 4:**
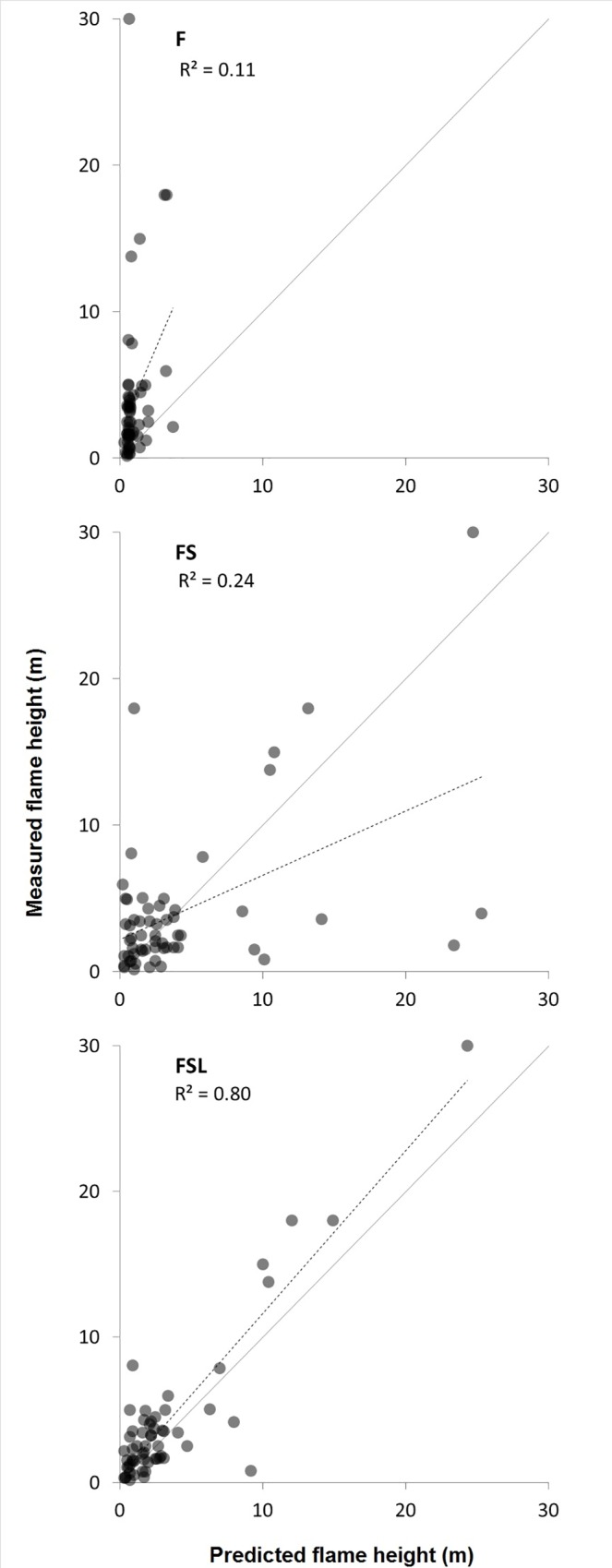
Median estimated vs. predicted flame height for the three treatments. Using surface fuel only (F), all predictions were for low flame heights (R^2^ = 0.11). Including structure with that fuel (FS) enabled the prediction of large flames, but the accuracy was low (R^2^ = 0.24). The inclusion of leaf traits (FSL) significantly improved the accuracy of predictions (R^2^ = 0.80), producing a MAE 3.80 times smaller than F, (p < 0.01, paired *t*-test), and 4.67 times smaller than FS (p < 0.01). The line of exact agreement is shown as solid, and the trend of the data is shown as a broken line, with R^2^ reported under the treatment name.

Observed fire severity ranged from surface (i.e. consumption of litter fuel only) at two sites to crown fire at four sites (Fig 4, S6 Table). Estimated flame heights ranged from 10 cm to more than 20 m with an average of 4.0 m (σ = 5.9 m). The range in estimated flame height varied between sites, with large levels of uncertainty evident when the gap between consumed vegetation strata and either the scorch height or the unconsumed strata above was large. The majority of sites (62%) were burnt by fires spreading either up or downslope with the wind behind them (usually head fires), 19% burnt downslope and against the wind (tail fires) and the remainder burnt either against the wind or across it, regardless of slope (flank fires).

### Flame height prediction

**Fig 5:**
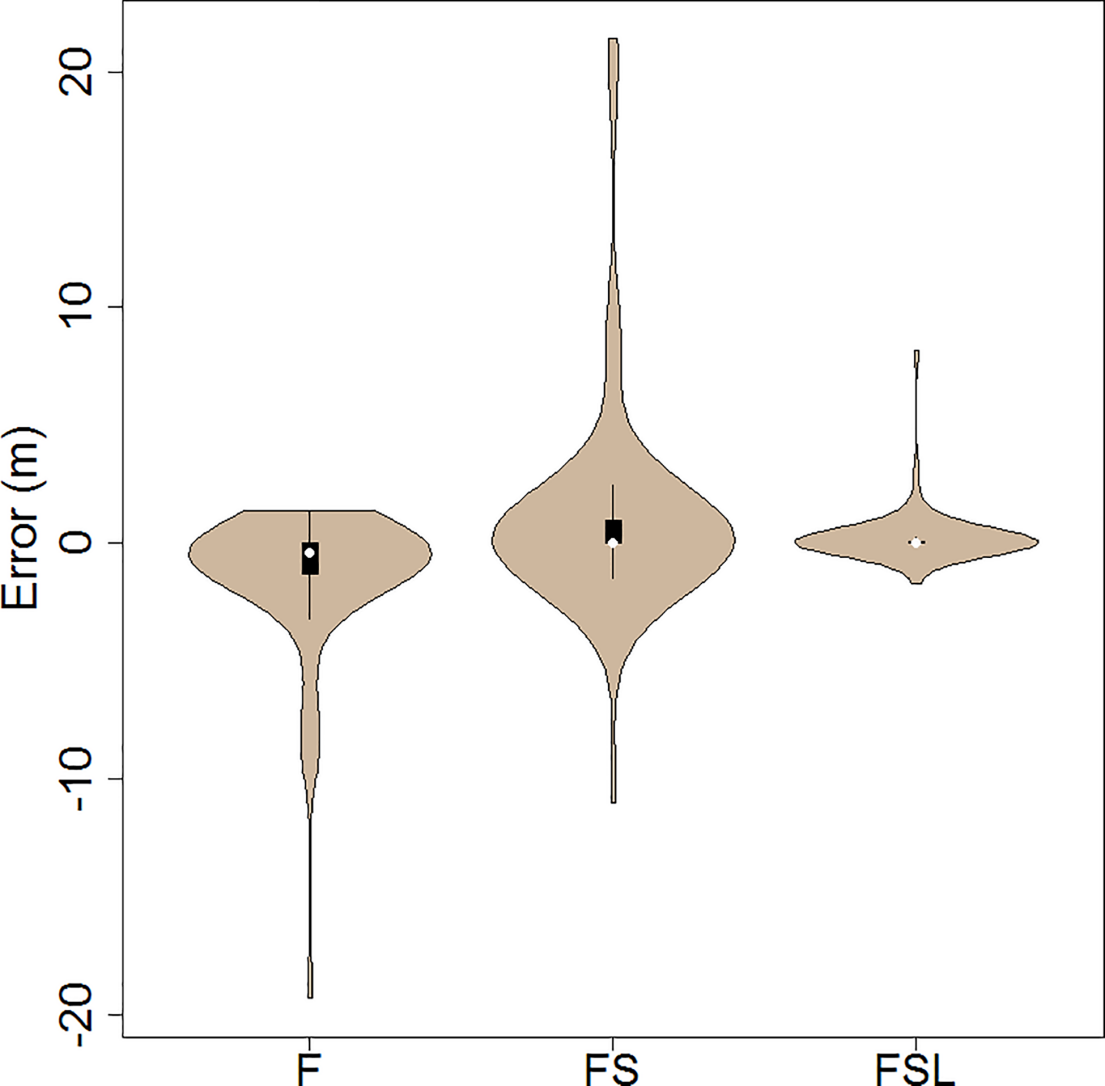
Violin plots showing error in flame height prediction for 58 plots modelled using three treatments of the FFM. As per [58], the box plots indicate data range, quartiles, and median, and the shaded area shows the density trace.

The successive inclusion of information on structure and other plant attributes improved the accuracy of predictions of flame dimensions relative to field estimates (Fig 4, Table 7). Flames predicted on the basis of surface fuel load only (F) were consistently small resulting in a large proportion (i.e. 31%) less than 1 m in height and a ME of -1.47 (Fig 5). As a result of the frequency of small observed flames, the MAE for these predictions was relatively low (1.53 m) despite the fact that it failed to predict any of the large flames that were observed.

**Table 7:** Mean Error and Mean Absolute Error (m) in flame height predictions for three model treatments of 58 sites, with standard error shown in brackets.

	F	FS	FSL
**ME**	-1.47 (0.43)	1.07 (0.59)	0.28 (0.16)
**MAE**	1.53 (0.42)	1.88 (0.56)	0.40 (0.15)

**Fig 6:**
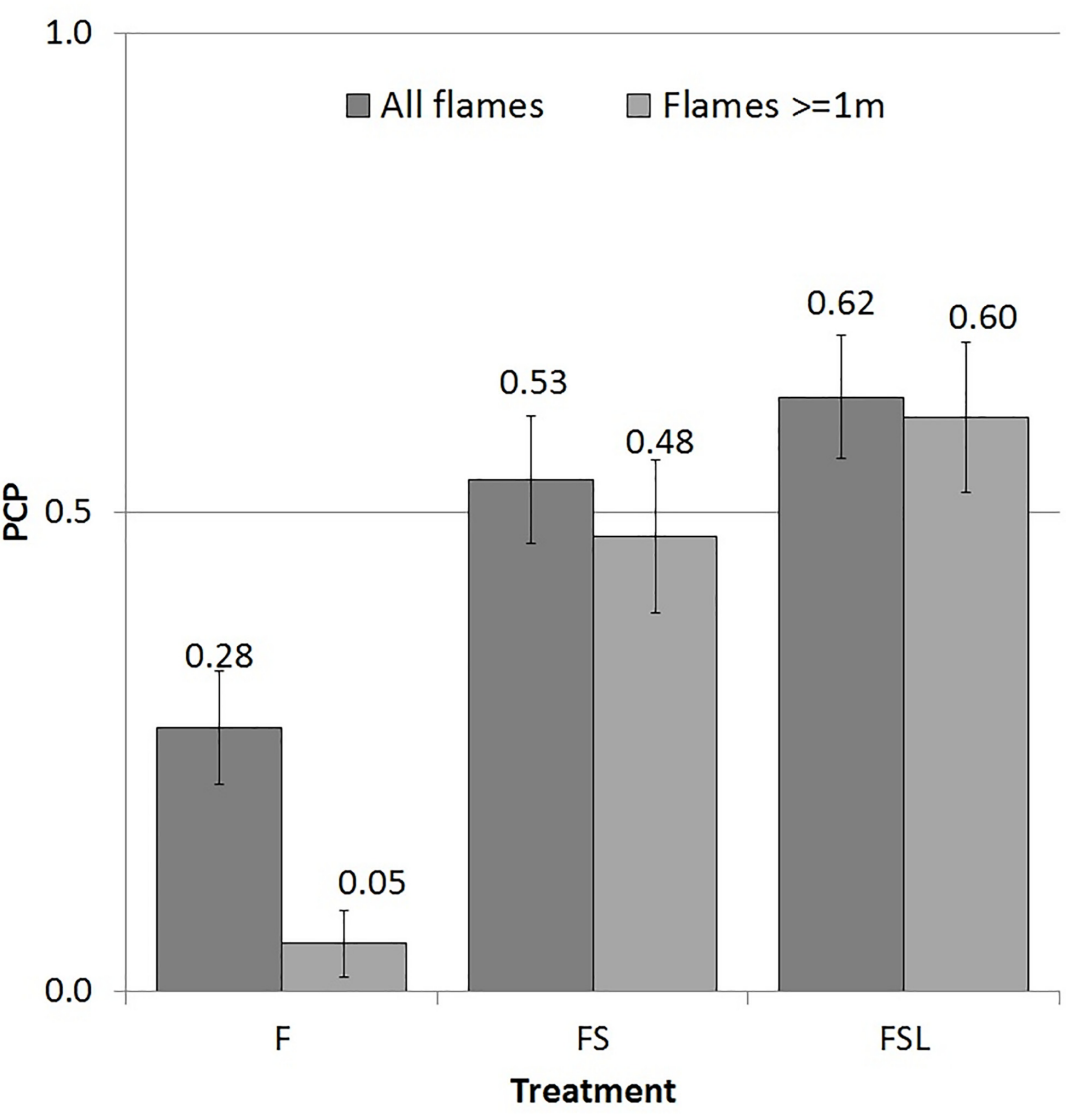
Proportion of correct predictions for each model treatment for the full flame height dataset (PCP) and for flames ≥1m (PCP_1_). PCP calculations were based on all 58 sites; PCP_1_ calculations were based on the 40 sites with flame heights ≥1m. Error bars show one standard error above and below the mean.

The inclusion of vegetation structural attributes without leaf trait information (FS) resulted in the prediction of large flames not previously predicted on the basis of F. However, in several sites where small flames were estimated, very large flames were predicted (Fig 4). As a result the MAE was higher for FS than for F (1.23 times the error, N.S., Fig 5). The PCP for FS was, however, 1.94 times higher than for F (p < 0.001, Fig 6), and PCP_1_ was 9.50 times higher (p < 0.001), which was indicative of the inability of F to predict these flames.

The accuracy of predictions was considerably improved with the inclusion of leaf traits (FSL), removing most of the false predictions of large flames (Fig 4). This provided an improvement of the MAE (0.40 m), which was 3.80 times more accurate than F (p < 0.01) and 4.67 times more accurate than FS (p < 0.01, Fig 5). PCP and PCP_1_ were respectively 2.25 and 12.00 times higher for FSL than for F (p < 0.001 for both), and 1.16, 1.26 times larger than for FS (p < 0.1 for both, Fig 6).

### Sensitivity of predicted flame height to environmental conditions and specific leaf traits

The most frequent (>70%) predictors included in the LASSO regression analyses were donor flammability (delta donor flame height), wind velocity, overstorey shelter (delta sum LAI) and dead fuel moisture content (DFMC); with delta canopy LAI and slope affecting the regression at moderate (>30%) frequency (Table 8). Receiver ignitability affected the predictions to only a minor degree (< 10%). Donor flammability (delta donor flame height) and overstorey shelter (delta LAI) were the most influential of these predictors, based on the size of the mean regression coefficients (Table 8). In comparison, effects of other influential predictors were weak, based on the size of their regression coefficients (Table 8)

**Table 8:** Regression coefficients for the predictors used in the LASSO regression, with lower (2.5%) and upper (97.5%) quantiles. Predictor groups are DF–donor flammability, RI–receiver ignitability, OS–overstorey shelter and General–exogenous, structural and surface parameters. Results are shown from 1000 regressions.

Predictor	Predictor Group	Number included	Mean regression coefficient	Lower quartile	Upper quartile
Delta donor Fh	DF	1000	2.28	2.16	2.67
Wind velocity	General	992	-0.02	-0.03	-0.01
DFMC	General	774	0.03	0.01	0.10
Delta sum LAI	OS	725	-0.11	-0.32	-0.03
Delta canopy LAI	OS	380	-0.27	-1.03	-0.06
Slope	General	380	0.01	0.00	0.03
Vertical continuity	General	136	1.34	0.07	3.18
Mean IC_FSL	RI	68	0.02	0.00	0.03
Donor Fh_FS	DF	64	-0.23	-0.30	-0.02
Canopy LAI_FS	OS	64	-2.98	-3.86	-0.34
Sum LAI_FS	OS	43	0.10	0.01	0.13
Surface fine fuel	General	31	0.00	0.00	0.00

Donor flammability, DFMC and slope had the effect of reducing flame height predicted using species leaf traits (FSL) relative to predictions based on structure only (FS) (i.e. positive regression coefficients, Table 8). By contrast, wind velocity and overstorey shelter (e.g. delta sum LAI) tended to increase predicted flame height when leaf traits were included (FSL) relative to predictions based on structure only (FS) (i.e. negative regression coefficients, Table 8).

## Discussion

Predictions of flame height in eucalypt forests were improved nearly four-fold when effects of plant community structure and species-level leaf traits were considered using the Forest Flammability Model, compared with use of surface-fuel load variables alone. The study therefore showed that floristic composition directly affects fire behaviour at the stand level through vegetation structure and leaf traits. As a result, changes to floristic composition have the potential to alter fire behaviour in a manner that cannot be accounted for by considering only conventional fuel parameters such as surface fuel load or understorey cover.

Variations in surface-fuel variables had almost no explanatory power for larger (≥1 m) flames but produced satisfactory predictions when flames were small. This suggests that surface fuel variables were important drivers of low intensity fires in these forests, but ignition of whole plants was the main cause of larger flames. Flame propagation across gaps between plants to produce larger flames was either limited or enabled by plant size and spacing and the flammability of leaves. Species-specific leaf traits are known to affect surface fire behaviour but models for representing these effects on the mass and packing of surface litter and subsequent fire behaviour are lacking. In addition, leaves of the *Eucalyptus* spp. that dominated the forests sampled in this study exhibited relatively small morphological variations (authors’ unpublished data). Thus we omitted consideration of effects of leaf variations on the flammability of surface litter in the current study Exploration of such effects will be an important priority for future research.

**Fig 7:**
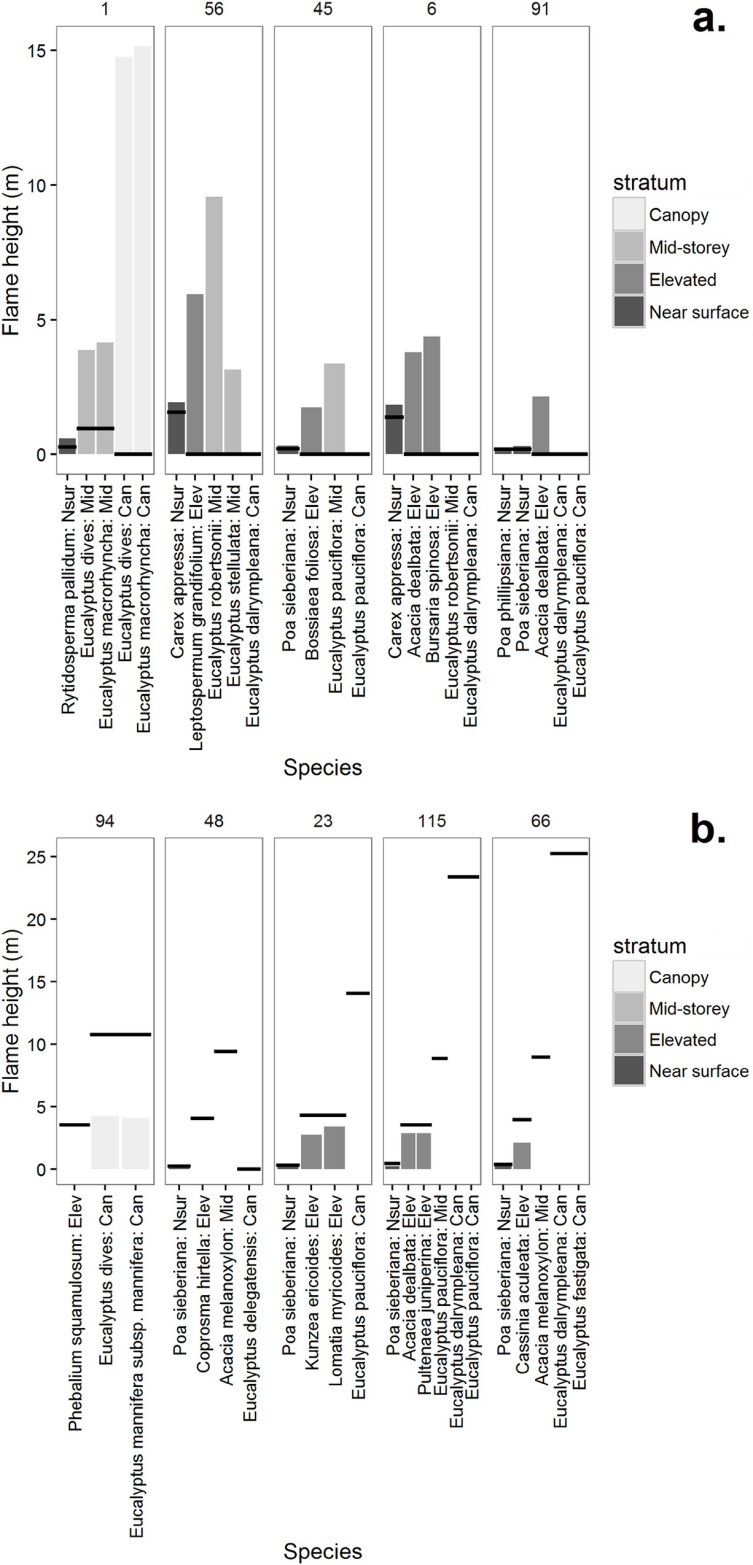
Donor flame height per species, stratum and site. Showing the five sites where the difference in prediction (FSL—FS) was most positive (a), and the five sites where it was most negative (b). Grey bars show flame heights per species from FSL and horizontal lines show flame heights from mean species in FS. Sites are ordered by delta flame height and the site number is given at the top of each plot.

Our analyses indicated that donor flame height was the most influential mechanism on overall predicted flame height (Fig 7). For example, predicted flame heights produced by FSL were substantially increased, relative to FS, from inclusion of specific leaf traits in sites 1, 56, 45, 6, 91 (Fig 7A). In these sites major increases in donor flame height in the near surface, elevated and midstorey layers were associated with increases in flame height of varying magnitude among a broad range of species (e.g. *Acacia*, *Bossiaea*, *Bursaria*, *Carex*, *Eucalyptus*, *Leptospermum* spp.) resulting from inclusion of specific leaf traits (Fig 7A). By contrast, in sites 94, 48, 23, 115, 56, predicted flame heights from FSL were substantially reduced relative to those predicted from FS (Fig 7B). Large reductions in donor flame height in many of the same species noted above, plus other species (*Coprosma*, *Kunzea*, *Lomatia*, *Phebalium*), resulting from the inclusion of leaf traits, were associated with the overall reductions in predicted flame height for these sites (Fig 7B). While these results illustrated the strong sensitivity of predicted flame height to leaf traits via changes to donor flammability, such effects were not evident for the same species and others in the majority of sites (S3 Fig). Expression of species-level effects was therefore conditional on other influences, including some of the other structural and environmental effects indicated in the ensemble regression analyses (Table 8).

Effects of overstorey sheltering (i.e. various measures of delta LAI, Table 8) on predicted flame height produced from FSL relative to FS were consistently negative. This indicated that LAI had a stronger dampening effect on overall predicted values when leaf traits were included (FSL) compared with FS. These trends possibly reflected the effects of inclusion of a much wider range of leaf sizes on wind penetration within and beneath plant canopies. Nonetheless the influence of delta LAI effects was small relative to that of donor flame height (Table 8).

As expected from previous analyses of fire severity variations in eucalypt forests [59–61], various aspects of weather influenced the sensitivity of predicted flame height. Wind velocity and DFMC were frequently influential because of the capacity to propagate flames across strata in the former case, and the availability of litter to burn in the latter case. Wind velocity had a positive influence on predicted flame height for FSL, so that flame height predictions were boosted for FSL compared with FS under stronger winds (Table 8). By contrast, increasing DFMC and slope tended to reduce flame height predicted by FSL relative to FS. In the models, DFMC not only affected litter but also suspended dead fuel in surface and elevated strata. DFMC in these above-ground layers may have been more influential on flame propagation when combined with leaf traits such as size and thickness. Effects of slope may have functioned similarly. In both cases, the magnitude of effects was weak (Table 8).

The net effect of the key mechanisms frequently included in the regression models was that a model not using leaf traits (i.e. FS) was more likely to over-predict flame height. This was exacerbated on steep slopes, when dead fuel was moister and when wind speeds were slower. In contrast, a model without leaf traits (FS) tended to under-predict flame height when LAI and consequent wind reduction were overestimated. Leaf traits therefore become increasingly important for predicting large flames under dry, windy conditions, and to a lesser extent, on shallow slopes.

Given the complexity of these mechanisms it was unsurprising that the sites exhibited a wide range of variation in comparative predictions of flame height (i.e. flame height FS–flame height FSL), including under-prediction and over-prediction (Fig 7) and relative insensitivity to the component species mix and abundances. Overall, variations in flame height predicted and validated in this study therefore emerged from complex interactions among species and traits. For example, fine leaves may be characterised as more flammable than thick leaves due to their shorter time to ignition [11,35,62]. The effect of this was apparent at site 45, where the receiver ignitability of fine leaves in the shrub *Bossiaea foliosa* was greater than the mean values used in FS, so that they ignited and thus propagated flames into the midstorey using FSL (Fig 7). Thinner leaves however also burn for less time [35], so that at site 66, the fine leaves of the shrub *Cassinia aculeata* reduced flame duration compared with mean values used in FS. This result contributed to a reduction in donor flame height (see S1 Table, S2 Fig and S4 Table for an explanation of the mechanism), preventing the upward propagation of flame into the higher strata using FSL (Fig 7). Thus the expression of basic patterns of flammability associated with particular leaf traits will be contingent on complex interactions among species and their three dimensional arrangement in any community. Higher order consequences of fundamental properties of flammability of particular plant traits may therefore not be readily predictable without detailed knowledge of the make-up of communities. The FFM provides a mechanistic understanding of such complexity and the resultant fire behaviour.

### Ecological implications

Our study found that for these sites, plant traits were more important for predicting flame height than was surface fuel load. This has important implications for fire ecology and management as it verifies some elements of a potential feedback relationship between fire behaviour and fire ecology that have been identified in conceptual models e.g. [63,64] but less commonly demonstrated and quantified in field studies. Invasive species such as grasses can alter fire regimes post-invasion [65] and differing plant communities can produce *in situ* micro-climates that are likely to alter the probability of fire e.g. [66]; however the ability of relatively subtle variations in species composition to differentially influence fire behaviour is less well explored.

While it is well known that particular fire regimes can select for certain species and thus alter the structure and composition of plant communities [67–69]; our study shows that variations in, for example, key shrub and herb species that may ensue from fire regime variations have the potential to influence subsequent fire behaviour via changes in plant traits and stand structure. Such effects on one aspect of fire behaviour—flame height, have the potential to cause flow-on effects on structure and composition via differential effects on survival and recovery of individuals of differing species. For example, the difference between a fire confined to surface or near-surface fuel strata versus a fire in the mid-storey or tree crowns may determine survival or mode of resprouting in various species of shrubs and trees in these forests [70,71]. Flame height will also have other important ecological influences in eucalypt forests, such as the nature of tree injuries and hollow formation, as well as the survival of arboreal mammals e.g. [72].

The results of the study indicate the need to more widely examine implications of forest structure and composition for fire behaviour. For example, the need to explore a wide range of plant trait variation in global vegetation models has been advocated [73,74]. Associated fire behaviour models may need to be structured in a way that more fully allows expression of the influences of such traits on flammability and fire behaviour.

### Model limitations and future development

An important limitation of the FFM is the time to ignition sub-model [35], which does not include the possible influences of leaf phosphate content or tissue density [11,13,62]. The FFM framework could incorporate such information once suitable relationships are developed and adequate data for individual species become widely available. The FFM utilises many inputs to describe the structure and leaf traits of component plant species, and this has the potential to constrain its use in operational applications. Nonetheless, the predictive accuracy of the current study was achieved using only some structural and leaf trait data measured in the field. Estimates for missing parameters were derived from photogrammetry, herbarium records and published databases e.g. [47]. Along with these approximations, satellite-based multi and hyper-spectral scanning offer possibilities for measuring parameters such as leaf moisture [63,64,75], particularly when supplemented with easily accessible information on species characteristics [76]. LiDAR has the potential to be used to measure structural parameters e.g. [77,78] and some leaf traits e.g.[79,80]. Further work should quantify model sensitivity to the accuracy of these inputs.

It is likely that a considerable proportion of the error in flame height predictions may be due the use of photogrammetric measurement of fuels, standard live moisture values, and the predictions of weather parameters from weather stations not located on the fire ground. Considerations of such sources of uncertainty and error will be a priority for future studies.

While we have demonstrated that the FFM more accurately predicted variations in flame length as a function of variations in the composition and structure of some eucalypt forests, further work is required to explore the accuracy of the FFM in other communities, and its capacity to represent other aspects of fire behaviour such as rate of spread. Such insights will help to determine the types of applications where the use of a complex model such the FFM may be most viable, given the demands of parametrisation of fuel compared with other fire behaviour models.

## Conclusions

The inclusion of species level traits in a modelling framework that represented flammability of plant parts and the resultant capacity of flames to propagate through their three dimensional arrangement (i.e. as represented in the FFM) considerably enhanced the ability to predict flame height in eucalypt forest communities. Conventional approaches to modelling fire behaviour based on the mass of surface litter and simple measures of above-ground fuel strata may therefore be unable to predict aspects of fire behaviour that arise from variations in forest composition. The FFM mechanistically explains the effects of plant traits on fire behaviour, thereby providing a basis for exploring the way in which variations in community composition can affect fire behaviour in the short-term and, perhaps, longer term fire regimes. The structure of the model also allows for the integration of new theoretical work on flammability and fire physics, providing an adaptive framework that can provide both direction for new research and application to evolutionary, ecological and management questions.

## Supporting Information

S1 Fig Processes in one time step. (PDF)

S2 Fig Example diagram. (PDF)

S3 Fig Donor flammability. (PDF)

S1 Table Model overview. (PDF)

S2 Table Sub-models used in the FFM for this study. (PDF)

S3 Table Ignition process in the FFM. (PDF)

S4 Table Plant and flame traits, and resultant heating as shown in S2 Fig. (PDF)

S5 Table Species leaf traits used in the FFM for this study. (PDF)

S6 Table Observed and predicted flame heights. (PDF)
