# Supplementary figures and images for: Biophysical Mechanistic Modelling Quantifies the Effects of Plant Traits on Fire Severity: Species, Not Surface Fuel Loads, Determine Flame Dimensions in Eucalypt Forests
Source: PLoS One. 2016 Aug 16;11(8):e0160715. doi: 10.1371/journal.pone.0160715 (PMC4986950; doi:10.1371/journal.pone.0160715)

## S1 Figure. Processes in one time step

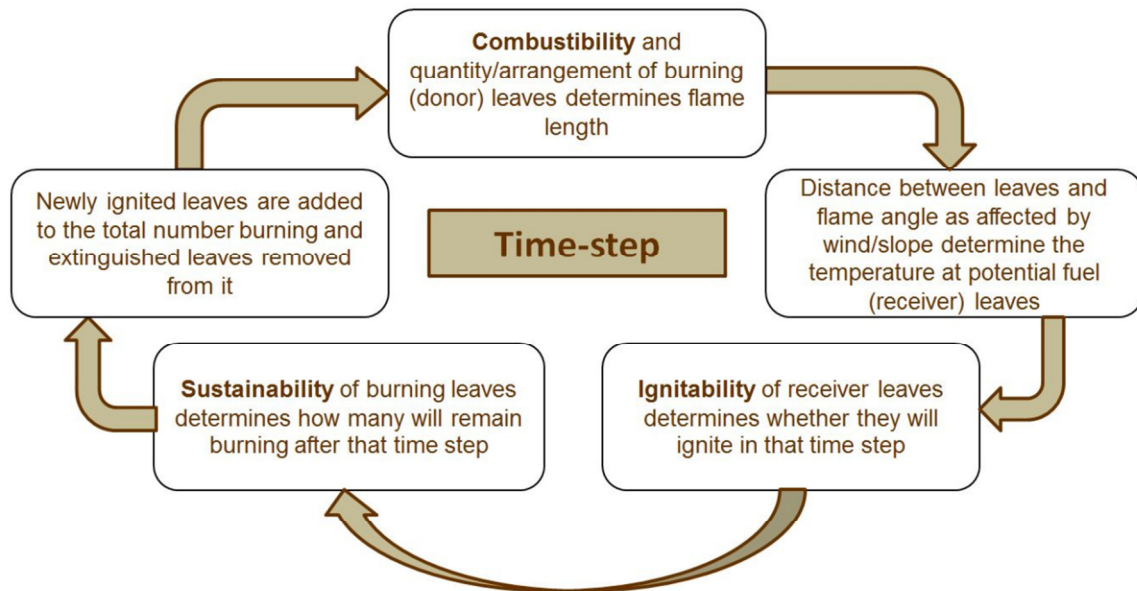

Supplement: S1 Fig — (PDF) [file pone.0160715.s001.pdf]
