# Supplementary material for: Biophysical Mechanistic Modelling Quantifies the Effects of Plant Traits on Fire Severity: Species, Not Surface Fuel Loads, Determine Flame Dimensions in Eucalypt Forests
Source: PLoS One. 2016 Aug 16;11(8):e0160715. doi: 10.1371/journal.pone.0160715 (PMC4986950; doi:10.1371/journal.pone.0160715)
Supplement: S2 Fig — (PDF) [file pone.0160715.s002.pdf]

S2 Figure. Example diagram

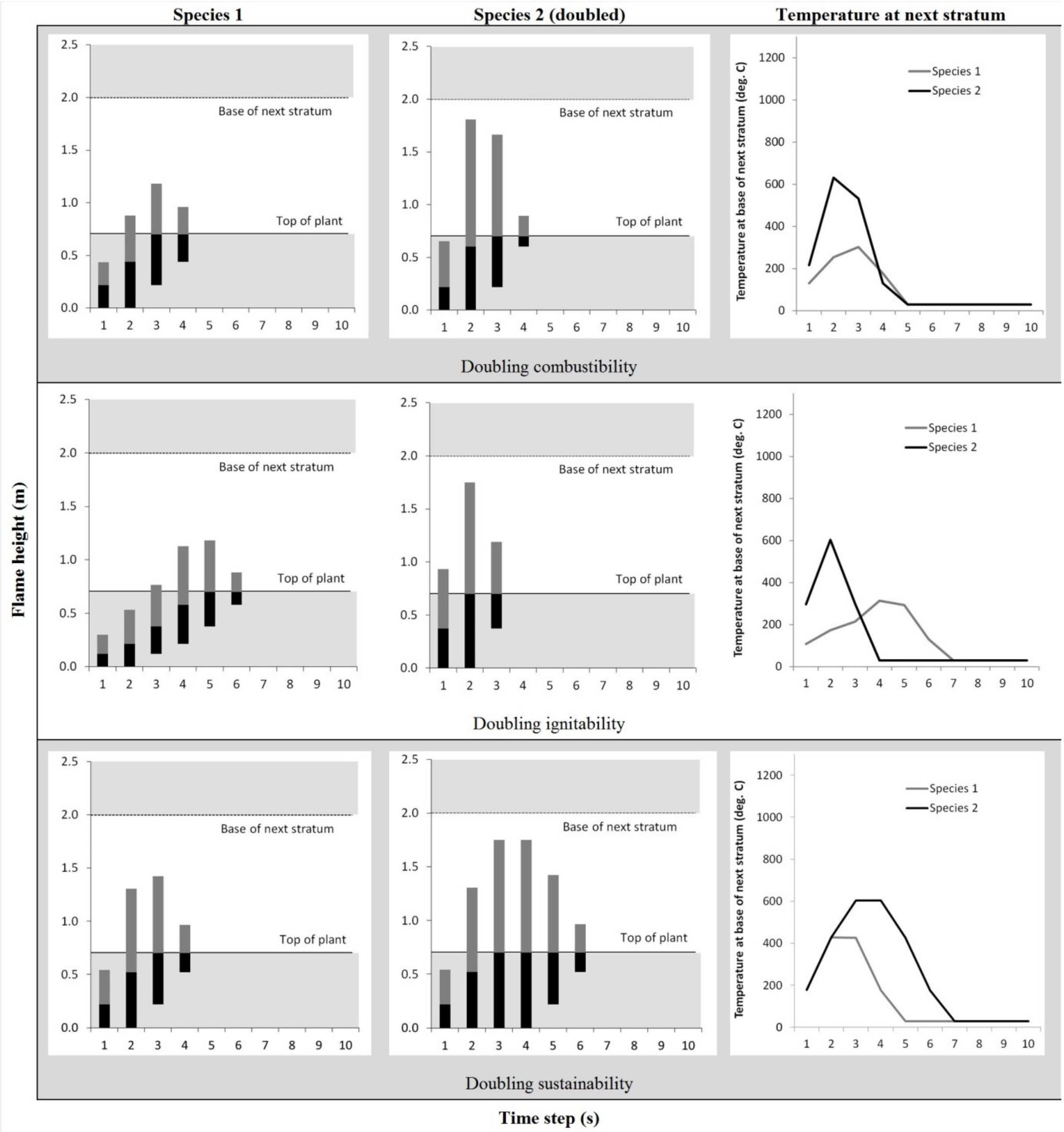

The effect of doubling different flammability metrics on flame height, flame duration and the temperature to which the base of next stratum is exposed. In each case, all metrics are held constant except for the one being examined. Shaded areas show plant strata, the dark bars show the part of the plant that is burning in that time step, and the lighter bars show the flame extending from the burning foliage.
