# Supplementary material for: Biophysical Mechanistic Modelling Quantifies the Effects of Plant Traits on Fire Severity: Species, Not Surface Fuel Loads, Determine Flame Dimensions in Eucalypt Forests
Source: PLoS One. 2016 Aug 16;11(8):e0160715. doi: 10.1371/journal.pone.0160715 (PMC4986950; doi:10.1371/journal.pone.0160715)
Supplement: S3 Fig — (PDF) [file pone.0160715.s003.pdf]

S3 Figure. Donor Flammability

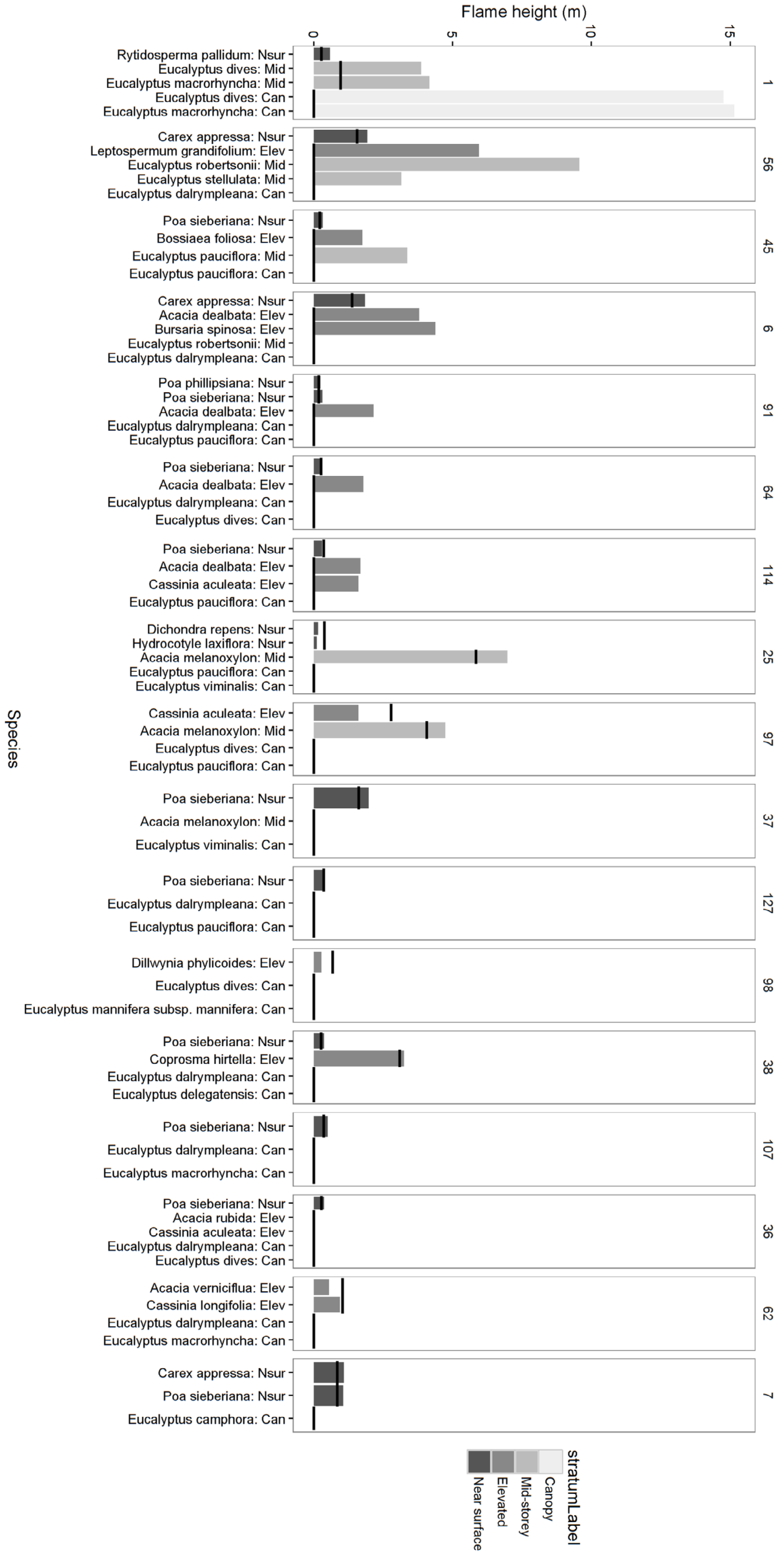

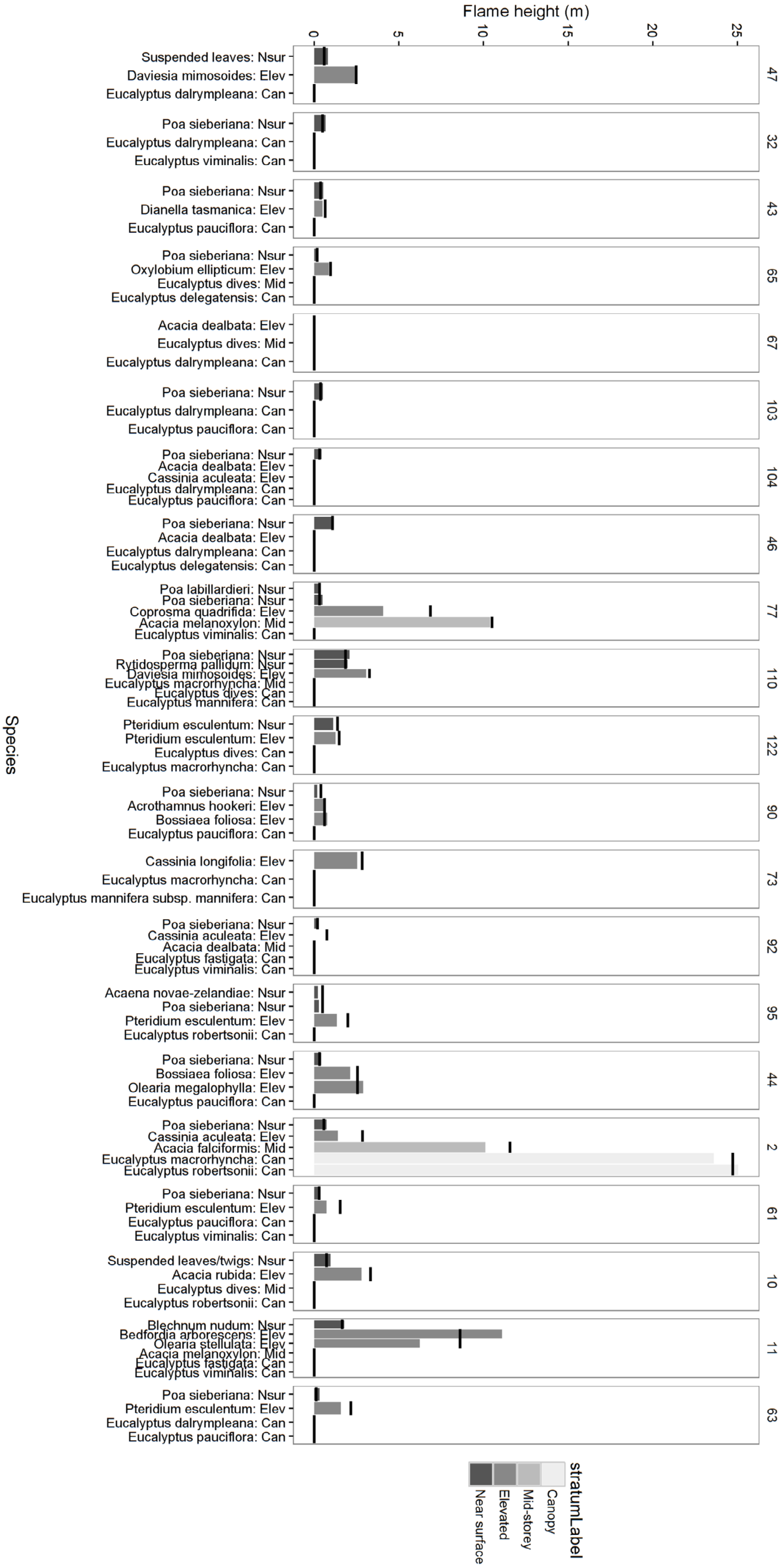
Flame height (m)
0
5
10
15
20
25
Species
Suspended leaves: Nsur
Daviesia mimosoides: Elev
Eucalyptus dalrympleana: Can
47
Poa sieberiana: Nsur
Eucalyptus dalrympleana: Can
Eucalyptus viminalis: Can
32
Poa sieberiana: Nsur
Dianella tasmanica: Elev
Eucalyptus pauciflora: Can
43
Poa sieberiana: Nsur
Oxylobium ellipticum: Elev
Eucalyptus dives: Mid
Eucalyptus delegatensis: Can
65
Acacia dealbata: Elev
Eucalyptus dives: Mid
Eucalyptus dalrympleana: Can
67
Poa sieberiana: Nsur
Eucalyptus dalrympleana: Can
Eucalyptus pauciflora: Can
103
Poa sieberiana: Nsur
Acacia dealbata: Elev
Cassinia aculeata: Elev
Eucalyptus dalrympleana: Can
Eucalyptus pauciflora: Can
104
Poa sieberiana: Nsur
Acacia dealbata: Elev
Eucalyptus dalrympleana: Can
Eucalyptus delegatensis: Can
46
Poa labillardieri: Nsur
Poa sieberiana: Nsur
Coprosma quadrifida: Elev
Acacia melanoxylon: Mid
Eucalyptus viminalis: Can
77
Poa sieberiana: Nsur
Rytidosperma pallidum: Nsur
Daviesia mimosoides: Elev
Eucalyptus macrorhyncha: Mid
Eucalyptus dives: Can
Eucalyptus mannifera: Can
110
Pteridium esculentum: Nsur
Pteridium esculentum: Elev
Eucalyptus dives: Can
Eucalyptus macrorhyncha: Can
122
Poa sieberiana: Nsur
Acrothamnus hookeri: Elev
Bossiaea foliosa: Elev
Eucalyptus pauciflora: Can
90
Cassinia longifolia: Elev
Eucalyptus macrorhyncha: Can
Eucalyptus mannifera subsp. mannifera: Can
73
Poa sieberiana: Nsur
Cassinia aculeata: Elev
Acacia dealbata: Mid
Eucalyptus fastigata: Can
Eucalyptus viminalis: Can
92
Acaena novae-zelandiae: Nsur
Poa sieberiana: Nsur
Pteridium esculentum: Elev
Eucalyptus robertsonii: Can
95
Poa sieberiana: Nsur
Bossiaea foliosa: Elev
Olearia megalophylla: Elev
Eucalyptus pauciflora: Can
44
Poa sieberiana: Nsur
Cassinia aculeata: Elev
Acacia falciformis: Mid
Eucalyptus macrorhyncha: Can
Eucalyptus robertsonii: Can
2
Poa sieberiana: Nsur
Pteridium esculentum: Elev
Eucalyptus pauciflora: Can
Eucalyptus viminalis: Can
61
Suspended leaves/twigs: Nsur
Acacia rubida: Elev
Eucalyptus dives: Mid
Eucalyptus robertsonii: Can
10
Blechnum nudum: Nsur
Bedfordia arborescens: Elev
Olearia stellulata: Elev
Acacia melanoxylon: Mid
Eucalyptus fastigata: Can
Eucalyptus viminalis: Can
11
Poa sieberiana: Nsur
Pteridium esculentum: Elev
Eucalyptus dalrympleana: Can
Eucalyptus pauciflora: Can
63
stratumLabel
Near surface
Elevated
Mid-storey
Canopy

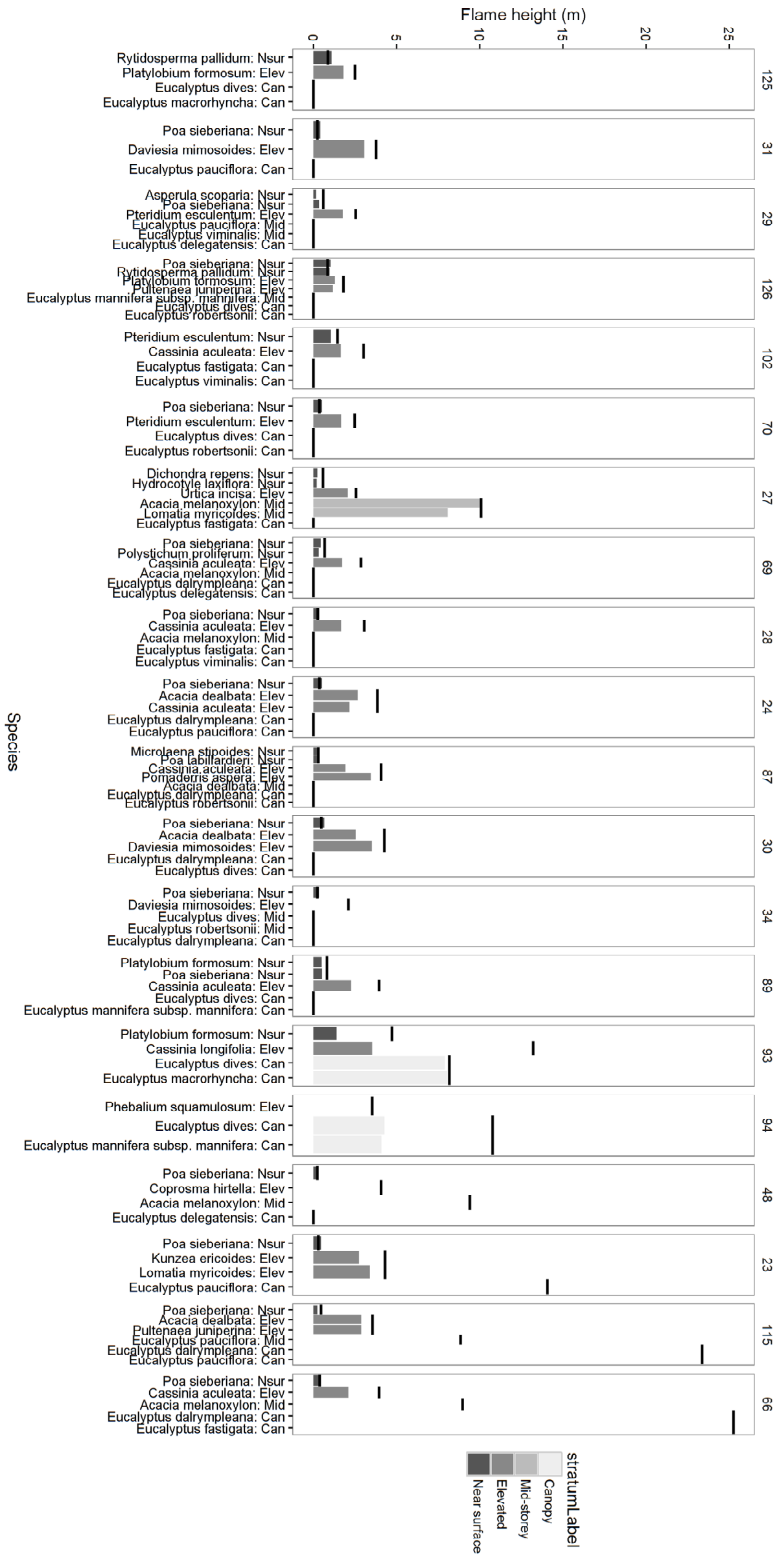

Donor flame height per species, stratum and site. Grey bars show flame heights per species from FSL and horizontal lines show flame heights from mean species in FS. Sites are ordered by delta flame height.
