# Supplementary material for: Biophysical Mechanistic Modelling Quantifies the Effects of Plant Traits on Fire Severity: Species, Not Surface Fuel Loads, Determine Flame Dimensions in Eucalypt Forests
Source: PLoS One. 2016 Aug 16;11(8):e0160715. doi: 10.1371/journal.pone.0160715 (PMC4986950; doi:10.1371/journal.pone.0160715)
Supplement: S1 Table — (PDF) [file pone.0160715.s004.pdf]

S1 Table. Model overview

**Model author**	Philip Zylstra<br>Centre for Environmental Risk Management of Bushfires,<br>University of Wollongong,<br>Wollongong NSW 2522<br>pzylstra@uow.edu.au.
**Coding**	Christopher Thomas<br>Michael Bedward
**Model code**	Free download of all code at<br>https://github.com/pzylstra/ffm_cpp/tree/Zylstra2016
**Input files**	Files numbered for site, with the suffix s for FS treatments, and a for FSL treatments.<br>https://github.com/pzylstra/ffm_cpp/tree/Zylstra2016/data
**Overview**	Full detail of the model cannot be communicated in this space; however the following pages provide a concise overview and the code can be accessed for detail of all equations and model mechanics.<br><br>The FFM is essentially a conceptual framework linking a series of sub-models (S2 Table), all of which can be replaced with other models of the same processes.<br><br>Fire spread is initiated using a surface fire behaviour model which works as a pilot flame. The process from this point is to establish whether flame can ignite fuel elements across the gaps that separate them, and if it does how this will change the fire behaviour. This is recalculated in one-second intervals or time-steps (S1 Figure), which work using conditional statements to analyse spread as a complex system (S3 Table).
**Scaling from leaf flammability to plant and stand flammability**	Plant flammability emerges from the ignitability, combustibility and sustainability of the component leaves, their density in the plant, the geometry of the plant and the conditions under which the plant is burning.<br><br>Ignitability is the combination of time to ignition TTI and the minimum temperature at which ignition can occur (E, below which TTI = ∞). TTI is rearranged in the model and combined with plume temperature to give the distance along a plume at which a leaf can be ignited within a time step. Relatively more ignitable leaves can ignite at lower temperatures further along the plume from the already burning fuel, which means that flame propagates faster through such plants.<br><br>Sustainability or flame residence in a leaf affects the number of leaves burning in a time step, as leaves with greater flame residence will continue to burn into future time steps, resulting in the accumulation of leaves that are alight.

	Leaf combustibility causes burning leaves to produce a flame of a given length λ_l, and the flame length at a given time step is the outcome of the number of burning leaves with individual flames coalescing into a single large flame (flame merging). Flame merging happens both laterally (M_{lat}) across a branch, a plant or between plants, and longitudinally (M_{lon} along the direction of the plume) within a branch or a plant.<br><br>S2 Figure shows these processes exemplified in a simplified form, where a 0.3m pilot flame ignites two plants with identical traits, except that in the three scenarios one aspect of flammability has been altered. In each case, the base height for the lower plant is 0.0 m, its top height is 0.7 m, and air temperature is modelled the base of next stratum at 2 m. Leaf and resulting flame traits are given in S4 Table, with flame traits given as the ratio sp.2:sp.1.<br><br>In all three scenarios, doubling the flammability trait resulted in changes to the heating of the next stratum; however the changes were not uniform. All increased the maximum temperature of heating, but the duration of flaming was changed in different ways – doubling combustibility produced no change, doubling ignitability decreased it by 50% and doubling leaf sustainability increased it by 50%.<br><br>These scenarios were greatly simplified because they used simple leaf flammability metrics rather than modelled ones, did not include pre-heating, and did not show the interaction of flame angle and plant shape. Some leaf traits have confounding effects on flammability; for example Zylstra's (2011) equations for TTI and FR_h both used leaf thickness, where thicker leaves took longer to ignite but burnt for longer.
