# Supplementary material for: Biophysical Mechanistic Modelling Quantifies the Effects of Plant Traits on Fire Severity: Species, Not Surface Fuel Loads, Determine Flame Dimensions in Eucalypt Forests
Source: PLoS One. 2016 Aug 16;11(8):e0160715. doi: 10.1371/journal.pone.0160715 (PMC4986950; doi:10.1371/journal.pone.0160715)
Supplement: S2 Table — (PDF) [file pone.0160715.s005.pdf]

S2 Table. Sub-models used in the FFM for this study

Code	Sub-model	Notes	Source
**FLAMMABILITY**			
TTI	Time to ignition	Predicted from temperature, moisture and leaf thickness	[1] Eq. 4.10
E	Endotherm	Entered as either measured value or modelled from silica-free ash content	[2] Eq. 1
FR_h	Flame residence in a heated leaf	Predicted from leaf dimensions and moisture	[1] Eq.4.21
FR_u	Flame residence in an unheated leaf	Cut-off temperature and default duration of 1s	-
FR_s	Flame residence in surface fuel	Predicted from mean fuel diameter	[3]
λ_l	Flame length from a leaf	Predicted from leaf dimensions and moisture	[1] Eqs. 4.18 - 4.20
T_{max}	Maximum flame temperature	Two values recorded in settings	[4,5]
L_n	Number of leaves per branch	Calculated from leaf separation, stem ramification and branch diameter	[1] Eq. 5.69
**FLAME PHYSICS**			
M_{lat}	Lateral merging of flames	Predicted from flame length and separation	[6]
L_{lon}	Longitudinal merging of flames	Predicted from flame length and depth ignited	[7]
θf_w	Effect of wind on flame angle	Predicted from flame length and wind speed	[8]
θf_s	Effect of slope on flame angle	Predicted for either point or line fires	[1] Eqs. 5.30, 5.33
θf_c	Interaction of wind and slope effects on flame angle	Cut-off value defines where competing effects dominate	-
θf_t	Effects of turbulence on flame angle	Set value defines a +/- value to apply to flame angle	-
λ_s	Surface flame length	Empirical model	[9]
ROS_s	Surface flame ROS	Empirical model	[9]
T_p	Plume temperature	Empirical model	[10]
**MICROCLIMATE**			
Ws	Wind profile	Empirical model	[1] Eq. 6.18 adjusted, [11–13]
T_a	Ambient temperature	Assumed equal throughout	-

References

1. Zylstra P. Forest Flammability: Modelling and Managing a Complex System [Internet]. University of NSW, Australian Defence Force Academy. 2011. doi:10.13140/2.1.3722.0166
2. Philpot CW. Influence of mineral content on the pyrolysis of plant materials. For Sci. 1970;16: 461–471.
3. Burrows ND. Flame residence times and rates of weight loss of eucalypt forest fuel particles. Int J Wildl Fire. 2001;10: 137–143.
4. Gould JS, Mccaw WL, Cheney NP, Ellis PF, Knight I, Sullivan AL. Project Vesta – fire in dry Eucalypt forest: fuel structure, fuel dynamics and fire behaviour. Canberra ACT, Perth WA; 2007.
5. Silvani X, Morandini F. Fire spread experiments in the field: Temperature and heat fluxes measurements. Fire Saf J. 2009;44: 279–285. doi:10.1016/j.firesaf.2008.06.004
6. Gill AM. Fire behaviour in discontinuous fuels: a contract report to the New South Wales National Parks and Wildlife Service. Canberra, ACT; 1990.
7. Mitler HE, Steckler KD. SPREAD - a model of flame spread on vertical surfaces. NISTIR 5619 [Internet]. Gaithersburg, MD; 1993. Available: http://fire.nist.gov/bfrlpubs/fire95/PDF/f95134.pdf
8. Van Wagner CE. Height of crown scorch in forest fires. Can J For Res. 1973;3: 373–378.
9. Burrows ND. Fire behaviour in Jarrah forest fuels: 1. Laboratory experiments. CALMScience. 1999;3: 31–56.
10. Weber RO, Gill AM, Lyons PRA, Moore PHR, Bradstock RA, Mercer GN. Modelling wildland fire temperatures. CALMScience Suppl. 1995;4: 23–26.
11. Cionco RM. A wind-profile index for canopy flow. Boundary-Layer Meteorol. 1972;3: 255–263.
12. Cionco RM. A mathematical model for air flow in a vegetative canopy. 1966. pp. 149–163.
13. Cionco RM. Analysis of canopy index values for various canopy densities. Boundary-Layer Meteorol. 1978;15: 81–93.
