# Supplementary material for: Biophysical Mechanistic Modelling Quantifies the Effects of Plant Traits on Fire Severity: Species, Not Surface Fuel Loads, Determine Flame Dimensions in Eucalypt Forests
Source: PLoS One. 2016 Aug 16;11(8):e0160715. doi: 10.1371/journal.pone.0160715 (PMC4986950; doi:10.1371/journal.pone.0160715)
Supplement: S3 Table — (PDF) [file pone.0160715.s006.pdf]

S3 Table. Ignition process in the FFM

Step	Action	Detail
IF	Flame can ignite new leaves across a gap	Distance is found geometrically from the gap between burning fuel and potential fuel, and the angle at which the plume crosses that gap.<br><br>Air temperature at the potential fuel is calculated using a T_p model for that distance, and the characteristics of the contributing flame that affect the T_p model.<br><br>Ignition occurs when TTI is ≤ 1 s (one time-step)
THEN	1. Add newly ignited fuel,<br>2. Remove newly consumed fuel<br>3. Calculate new flame traits	Add the number of leaves that were ignited to those already burning and remove the leaves that have been burning for at least the period of their calculated flame duration.<br><br>Calculate the flame length from individual burning leaves, then the merged flame length from all of these combined.<br><br>Find the wind speed at that point, and then the angle of the flame.
ELSE	1. Treat partial drying of leaves that failed to ignite as pre-heating<br>2. Remove newly consumed fuel<br>3. Calculate new flame traits	Reduce the TTI of the leaves by the amount dried.<br><br>Remove from the total of leaves already burning those that have been burning for at least the period of their calculated flame duration.<br><br>Calculate the flame length from individual burning leaves, then the merged flame length from all of these combined.<br><br>Find the wind speed at that point, and then the angle of the flame.
