# Supplementary material for: Biophysical Mechanistic Modelling Quantifies the Effects of Plant Traits on Fire Severity: Species, Not Surface Fuel Loads, Determine Flame Dimensions in Eucalypt Forests
Source: PLoS One. 2016 Aug 16;11(8):e0160715. doi: 10.1371/journal.pone.0160715 (PMC4986950; doi:10.1371/journal.pone.0160715)
Supplement: S4 Table — (PDF) [file pone.0160715.s007.pdf]

S4 Table. Plant and flame traits, and resultant heating as shown in S2 Figure.

	Leaf traits			Flame traits and heating		
	**Ignitability** (Min. temp for ignition within 1 s, °C)	**Combustibility** (Ratio of flame extension to depth ignited)	**Sustainability** (Flame duration, s)	**Max flame height ratio**	**Flame duration ratio**	**Max temp at next stratum ratio**
Row 1	1000	1, 2	2	1.5	1.0	2.1
Row 2	600, 1200*	1.5	2	1.5	0.5	1.9
Row 3	1000	1.5	2, 4	1.2	1.5	1.4

*Where two numbers are given, the first is for sp. 1 and the second for sp. 2.
