# Supplementary material for: Biophysical Mechanistic Modelling Quantifies the Effects of Plant Traits on Fire Severity: Species, Not Surface Fuel Loads, Determine Flame Dimensions in Eucalypt Forests
Source: PLoS One. 2016 Aug 16;11(8):e0160715. doi: 10.1371/journal.pone.0160715 (PMC4986950; doi:10.1371/journal.pone.0160715)
Supplement: S5 Table — (PDF) [file pone.0160715.s008.pdf]

S5 Table. Species leaf traits used in the FFM for this study

Species	Thickness (mm)	Moisture (%ODW)	Endotherm (deg. C)	Width (mm)	Length (mm)	Ramification	Leaf separation (cm)	Mean clump width (cm)	Range	Mean clump separation (cm)	Range
Acacia dealbata subsp. *subalpina*	0.22[a]	100	220[a]	0.5[a]	2.7[a]	4.8[a]	0.18[a]	60	83	18	22
Acacia falciformis	0.30	100	260	28.2	102.3	4.6	1.35	142	0	91	0
Acacia melanoxylon	0.21	150	260	11.0	95.4	5.6	1.60	135	236	35	45
Acacia rubida	0.36	100	260	14.2	87.3	2.4	1.50	46	0	38	0
Acacia verniciflua	0.26	100	260	4.1	24.5	2.8	1.62	93.8	0	31.3	0
Acaena novae-zelandiae	0.09	100	260	3.9	7.2	3.0	0.24	16.8	0	0.0	0
Acrothamnus hookeri	0.22	100	260	1.7	6.3	4.6	0.14	7.7	0	4.9	0
Asperula scoparia	0.11	150	260	0.5	2.7	4.0	0.11	10.0	0	0.0	0
Bedfordia arborescens	0.28[a]	200	260	25.8[a]	136.6[a]	3.0[a]	0.78[a]	79.5	0	11.4	0
Blechnum nudum	0.20	200	260	14.1	128.6	1.0	0.43	19.4	0	8.3	0
Bossiaea foliosa	0.17[a]	100	300[a]	5.0[a]	5.0[a]	5.0[a]	0.10[a]	29	56	6	3
Bursaria spinosa	0.24	100	260	4.3	14.8	4.5	0.25	95	0	11	0
Carex appressa	0.30	100	260	11.0	150.0	1.0	2.00	93	60	0	0
Cassinia aculeata	0.29	100	220	0.5	9.4	4.8	0.18	50	60	19	29
Cassinia longifolia	0.17	100	220	4.0	50.2	5.0	0.42	70	40	27	7
Coprosma hirtella	0.67	100	260	20.9	34.1	5.2	0.71	78	74	22	19
Coprosma quadrifida	0.14	100	260	3.6	6.3	5.6	0.21	88	0	32	0
Daviesia mimosoides subsp. *mimosoides*	0.38[a]	100	280[a]	10.8[a]	58.2[a]	2.2[a]	1.26[a]	68	44	19	7
Dianella tasmanica	0.44	150	260	6.4	150.0	1.0	15.30	27.6	0.0	0.0	0.0
Dichondra repens	0.30	150	260	11.0	18.9	1.0	0.56	11	18	3	5
Dicksonia antarctica	0.10	200	260	2.5	4.5	3.0	0.15	286	0	36	0
Dillwynia phylicoides	0.22	100	260	1.7	5.5	3.6	0.14	18	0	7	0
Eucalyptus camphora subsp. *humeana*	0.34	100	220	49.8	94.0	5.5	3.23	400	0	234	0
Eucalyptus dalrympleana subsp. *dalrympleana*	0.41	100	220	20.5	134.0	5.0	1.83	328	506	110	139
Eucalyptus delegatensis subsp. *delegatensis*	0.37[a]	100	220	29.7[a]	131.6[a]	5.4[A]	2.48[A]	404	448	120	101
Eucalyptus dives	0.40[B]	100	220	15.8[B]	103.6[B]	5.0	1.66	250	380	98	123
Eucalyptus fastigata	0.34	100	220	23.8	96.0	5.0	0.58	409	143	144	68
Eucalyptus macrorhyncha	0.40	100	220	16.5	80.2	6.0	1.17	206	296	90	161
Eucalyptus mannifera subsp. *mannifera*	0.39	100	220	7.6	118.0	4.8	1.22	227	238	110	141
Eucalyptus pauciflora	0.49[a]	100	220	26.7[a]	67.9[a]	5.0[a]	1.06[a]	233	386	106	205
Eucalyptus robertsonii subsp. *robertsonii*	0.23	100	220	16.0	80.9	5.5	0.60	255	393	119	196
Eucalyptus stellulata	0.43[a]	100	220	20.6[a]	59.4[a]	6.4[a]	1.41[a]	92	0	30	0
Eucalyptus viminalis	0.38	100	220	10.6	59.8	5.5	1.24	333	268	133	103
Hydrocotyle laxiflora	0.27	150	260	17.4	19.7	1.0	5.00	11	18	3	5
Kunzea ericoides	0.22	100	220	2.6	10.1	4.8	0.20	86	0	14	0
Leptospermum grandifolium	0.25	100	220	3.9	10.6	5.2	0.12	184	0	71	0

Lomatia myricoides	0.25	100	260	4.4	91.9	3.0	1.07	95	19	20	12
Microlaena stipoides	0.15[c]	150	260	3.9	150.0	1.0	1.58	30	0	0	0
Olearia megalophylla	0.56	100	260	12.8	44.1	3.2	0.53	64	0	8	0
Olearia stellulata	0.23	100	260	4.1	11.1	2.6	0.19	80	0	11	0
Oxylobium ellipticum	0.28	100	260	9.0	21.2	3.8	0.36	41	0	4	0
Phebalium squamulosum subsp. ozothamnoides	0.31	100	260	1.6	11.1	4.8	0.17	88	0	19	0
Platylobium formosum	0.31	100	260	24.6	34.9	1.6	1.99	26	21	7	13
Poa helmsii	0.16	100	350[d]	1.3	150.0	1.0	0.38	130	0	0	0
Poa labillardierei	0.25	100	350[d]	1.3	150.0	1.0	0.92	28	5	0	0
Poa phillipsiana	0.17	100	350[d]	0.5	150.0	1.0	0.44	13	0	0	0
Poa sieberiana	0.16	100	350[d]	1.3	150.0	1.0	0.38	21	87	0	0
Polystichum proliferum	0.24	200	347[d]	5.0	11.3	2.0	0.33	17	0	0	0
Pomaderris aspera	0.31[a]	100	260	13.1[a]	42.2[a]	4.0[a]	1.67[a]	51	0	18	0
Pteridium esculentum	0.21	100	260	2.2	8.7	4.0	0.20	31	6	14	3
Pultenaea juniperina	0.23	100	260	1.2	5.5	6.4	0.07	43	10	13	0
Rytidosperma pallidum	0.34	100	260	2.4	150.0	1.0	0.78	74	34	0	0
Urtica incisa	0.21	200	260	55.5	62.5	2.2	2.35	10	0	15	0

Data collected as part of this study except where indicated by the following superscripts: [a][1], [b][2], [c][3,4], [d][5], using [6]

References

1. Zylstra P. Forest Flammability: Modelling and Managing a Complex System [Internet]. University of NSW, Australian Defence Force Academy. 2011. doi:10.13140/2.1.3722.0166
2. Gill AM, Moore PHR. Ignitability of leaves of Australian plants. Contract report to the Australian flora foundation. Canberra, ACT; 1996.
3. Kattge J, Díaz S, Lavorel S, Prentice IC, Leadley P, Bönisch G, et al. TRY - a global database of plant traits. Glob Chang Biol. 2011;17: 2905–2935. doi:10.1111/j.1365-2486.2011.02451.x
4. Onoda Y, Westoby M, Adler PB, Choong AMF, Clissold FJ, Cornelissen JHC, et al. Global patterns of leaf mechanical properties. Ecol Lett. 2011;14: 301–312. doi:10.1111/j.1461-0248.2010.01582.x
5. Dickinson K, Kirkpatrick JB. The flammability and energy content of some important plant species and fuel components in the forests of southeastern Tasmania. J Biogeogr. 1985;12: 121–134. Available: http://www.jstor.org/discover/10.2307/2844836?uid=2&uid=4&sid=21105117046943
6. Philpot CW. Influence of mineral content on the pyrolysis of plant materials. For Sci. 1970;16: 461–471.
