# Supplementary material for: Biophysical Mechanistic Modelling Quantifies the Effects of Plant Traits on Fire Severity: Species, Not Surface Fuel Loads, Determine Flame Dimensions in Eucalypt Forests
Source: PLoS One. 2016 Aug 16;11(8):e0160715. doi: 10.1371/journal.pone.0160715 (PMC4986950; doi:10.1371/journal.pone.0160715)
Supplement: S6 Table — (PDF) [file pone.0160715.s009.pdf]

S6 Table. Observed and predicted flame heights

	Exogenous conditions			Flame height (m)				
				Observations		Treatments		
SITE	Slope (degrees)	Wind velocity (km/h)	DFMC	Minimum	Maximum	F	FS	FSL
1	18.1	19.3	3.6%	12.0	24.0	3.2	1.0	14.9
2	-2.3	21.0	3.6%	20.0	40.0	0.7	24.7	24.3
6	-0.8	-1.3	7.3%	2.9	4.0	0.6	1.4	4.1
7	-1.0	-3.4	7.2%	0.5	15.6	0.6	0.8	0.9
10	-5.2	6.2	7.2%	1.4	2.0	0.5	3.3	2.8
11	1.7	18.5	3.6%	3.3	5.0	0.7	8.6	8.0
23	0.0	-13.1	3.6%	2.0	5.2	0.5	14.1	3.0
24	0.0	-13.1	3.6%	2.0	5.5	0.7	3.8	2.4
25	-11.0	22.4	3.6%	6.0	9.7	0.9	5.8	7.0
27	10.0	-5.5	7.6%	0.7	1.0	0.7	10.1	9.2
28	-0.7	1.1	8.7%	1.2	2.0	0.7	3.1	1.7
29	-23.0	10.4	7.4%	1.0	4.0	0.7	2.5	1.8
30	17.3	-20.6	8.8%	2.0	3.0	0.5	4.3	2.7
31	0.0	5.4	7.6%	1.4	2.0	0.7	3.8	3.1
32	5.1	-14.8	13.7%	0.2	3.0	0.9	0.9	0.9
34	-7.8	-7.2	7.1%	0.1	0.5	0.7	2.1	0.4
36	-13.2	2.7	7.6%	0.1	0.7	0.4	0.3	0.4
37	8.5	-0.4	7.6%	1.0	1.8	0.6	1.6	2.0
38	-2.1	1.2	7.2%	2.0	8.0	0.6	3.1	3.2
43	0.0	-5.7	3.7%	0.3	6.0	0.7	0.7	0.7
44	-4.7	4.9	3.6%	2.0	4.5	0.7	2.6	2.2
45	10.8	27.5	3.7%	3.0	8.9	3.2	0.2	3.4
46	-17.9	1.5	8.2%	0.1	0.9	0.6	1.1	1.0
47	-13.0	-0.6	8.3%	1.3	2.0	0.5	2.5	2.5
48	-12.2	11.3	12.4%	1.1	2.0	0.7	9.4	0.5
56	-0.6	5.2	13.5%	4.0	6.0	0.6	1.6	6.3

61	17.2	10.3	13.6%	1.0	2.0	1.2	1.5	0.9
62	-22.1	-13.7	11.7%	0.0	0.4	0.5	1.0	0.7
63	0.0	9.0	13.5%	1.0	5.9	0.8	2.1	1.6
64	1.0	21.5	3.6%	2.5	7.4	1.6	0.5	1.8
65	-28.5	-5.6	3.6%	1.0	6.1	0.6	1.0	0.9
66	-0.5	17.5	3.6%	2.0	6.0	0.7	25.3	2.1
67	-8.6	-14.7	5.9%	0.0	0.7	0.5	0.3	0.3
69	-1.6	-5.9	3.6%	0.1	0.6	0.7	2.9	1.7
70	11.6	-20.6	3.6%	1.2	3.0	0.6	2.5	1.7
73	0.0	18.1	3.6%	2.0	7.0	1.4	2.8	2.5
77	3.6	23.9	3.6%	8.0	19.6	0.8	10.5	10.4
87	0.3	-3.9	7.2%	1.3	2.0	0.6	4.1	2.6
89	8.5	-12.0	3.6%	2.0	6.5	0.6	3.9	2.2
90	0.0	19.8	7.6%	0.5	2.0	1.9	1.0	0.7
91	20.7	28.7	3.6%	2.5	4.0	2.0	0.4	2.2
92	14.3	25.7	3.6%	2.0	2.3	3.7	0.7	0.3
93	17.9	30.2	3.6%	12.0	24.0	3.3	13.2	12.0
94	-5.0	25.5	3.6%	10.0	20.0	1.4	10.8	10.0
95	-18.0	25.5	3.6%	1.0	7.7	1.0	2.0	1.7
97	-1.4	24.8	3.6%	2.0	3.0	2.0	4.1	4.7
98	0.0	15.9	3.6%	0.5	4.1	1.3	0.8	0.9
102	-13.0	0.9	8.8%	1.4	2.5	0.7	3.0	1.6
103	2.8	-18.3	7.6%	0.1	2.0	0.6	0.6	0.6
104	6.6	-19.6	7.6%	0.1	1.3	0.7	0.7	0.7
107	0.5	-6.4	13.7%	0.1	2.0	0.3	0.3	0.5
110	14.6	8.7	13.0%	2.0	5.1	0.7	3.3	3.1
114	4.0	20.2	7.6%	0.1	1.4	1.4	0.8	1.6
115	13.5	12.1	7.3%	1.6	2.0	1.0	23.4	2.9
122	13.9	7.8	6.8%	1.0	4.0	0.8	1.5	1.2
125	20.8	2.7	8.3%	0.5	1.0	0.7	2.5	1.8
126	-3.1	-4.5	6.9%	1.0	2.0	0.6	1.8	1.0
127	1.5	25.2	10.8%	0.1	9.8	1.8	0.4	0.7
